# LincRNA-miR interactions in hepatocellular carcinoma: comprehensive review and in silico analysis: a step toward ncRNA precision

**DOI:** 10.1007/s00210-025-04285-7

**Published:** 2025-05-23

**Authors:** Nadia M. Hamdy, Al-Aliaa M. Sallam, Ola Elazazy, Ahmed M. Kabel, Rania M. Salama, Shaimaa A. Gouhar, Sherien M. El-Daly, Samar F. Darwish

**Affiliations:** 1 Biochemistry Department, Faculty of Pharmacy, Ain Shams University, Abassia, Cairo, 11566 Egypt; 2 Biochemistry Department, Faculty of Pharmacy, Badr University in Cairo (BUC), Badr City, 11829 Cairo Egypt; 3 Department of Pharmacology, Faculty of Medicine, Tanta University, Tanta, Egypt; 4 Pharmacology and Toxicology Department, Faculty of Pharmacy, Misr International University (MIU), Cairo, Egypt; 5 Medical Biochemistry Department, Medical Research and Clinical Studies Institute, National Research Centre, Giza, 12622 Egypt; 6 Cancer Biology and Genetics Laboratory, Centre of Excellence for Advanced Sciences, National Research Centre, Giza, 12622 Egypt; 7 Pharmacology and Toxicology Department, Faculty of Pharmacy, Badr University in Cairo (BUC), Badr City, 11829 Cairo Egypt

**Keywords:** NcRNA, LncRNAs, LincRNAs, Liver cancer, In silico analysis, Precision medicine

## Abstract

The most prevalent form of primary liver cancer and one of the chief drivers of cancer-related mortality globally is hepatocellular carcinoma (HCC). Imminent evidence has indicated that non-coding RNAs (ncRNAs) play an integral part in the development and propagation of HCC. RNA stabilization, transcription regulation, chromatin and genomic architecture remodeling, enhancer-associated activity, and other varied properties set long intergenic ncRNA (lincRNA) genes apart from messenger RNA (mRNA)-encoding genes. Through a variety of processes, lincRNAs may generally be used to fine-tune the transcription of nearby genes with exceptional tissue specificity, underscoring our quickly developing knowledge of the non-coding genome. Through their binding with divergent cell targets, some HCC-related ncRNAs have been demonstrated to exhibit abnormal expression, contribute to malignant growth, evade apoptosis, and have invasive potential. Therefore, a better comprehension of lincRNA dysregulation might offer novel perspectives on the pathophysiology of HCC as well as innovative instruments for the early detection and management of HCC. In the present review, we provide an overview of the increasing relevance of lincRNAs as a major contributor to the pathophysiology of HCC, emphasizing their influence on signaling pathways implicated in the development, progression, and response to treatment of tumors. In addition, we go over the new approaches that target lincRNAs for HCC treatment as well as the possible therapeutic uses of lincRNAs as prognostic and diagnostic biomarkers for HCC.

**Graphical Abstract:**

The multifaceted roles of long intergenic non-coding RNAs (lincRNAs) in hepatocellular carcinoma (HCC).

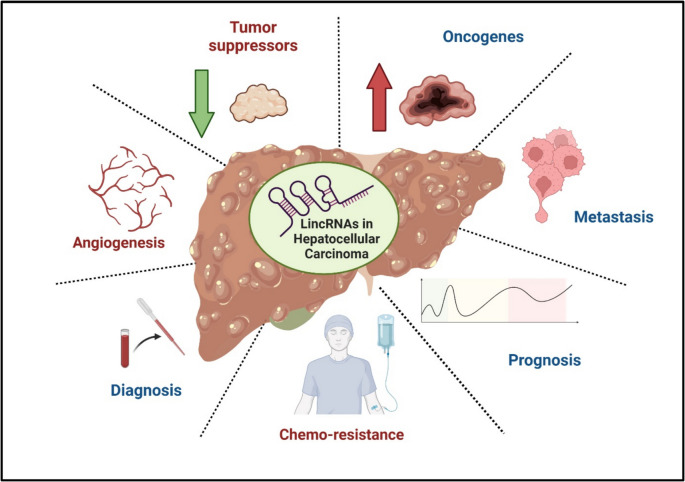

**Supplementary Information:**

The online version contains supplementary material available at 10.1007/s00210-025-04285-7.

## Introduction

### Background

The most prevalent type of primary liver cancer that develops from hepatocytes is hepatocellular carcinoma (HCC). According to Global Cancer Incidence, Mortality and Death Statistics, it is the third leading cause of cancer-related deaths worldwide and the sixth most frequent type of cancer prevalence (GLOBOCAN 2022) (Bray et al. 2024). It is defined by its aggressiveness and lack of available treatments, and it primarily arises in the setting of chronic liver disease. HCC is a serious worldwide health issue that varies greatly by region. The highest incidence rates are found in areas like Eastern and South-Eastern Asia, Northern and Western Africa, and sub-Saharan Africa where chronic hepatitis B virus (HBV) and hepatitis C virus (HCV) infections that cause HCC are endemic (El-Mesallamy et al. 2011; Shah and Sarkar 2024). Aflatoxin exposure, non-alcoholic fatty liver disease (NAFLD) which has been called metabolic dysfunction-associated fatty liver disease (MAFLD), and alcoholic liver disease are additional important risk factors (Eslam et al. 2020). Understanding the molecular pathways causing HCC has been a prominent focus of research due to the heterogeneous nature of its etiology (Bray et al. 2024). Gene mutations and other genetic and epigenetic changes are frequent in HCC (Rebouissou and Nault 2020). Moreover, the formation and progression of HCC are significantly influenced by the deregulation of important signaling networks, including the phosphoinositide 3-kinase (PI3 K)/protein kinase B (AKT), mitogen-activated protein kinase (MAPK), and wingless-related integration site (Wnt)/β-catenin pathways. The complex interactions between these pathways and their potential as therapeutic targets have been clarified by recent studies (Llovet et al. 2022).

### Long non-coding RNAs

Long non-coding RNAs (lncRNAs) are a class of non-protein-coding transcripts longer than 200 nucleotides that have been recently identified and extensively studied. To date, more than 50,000 lncRNAs have been discovered, representing over 60% of the human transcriptome (Fang et al. 2018). Furthermore, the role of lncRNAs in physiological and pathological processes has been substantiated by a wealth of experimental data. Specifically, both oncogenic and tumor-suppressive lncRNAs have been identified across multiple cancer types, including HCC (Xie et al. 2021).

Similar to mRNAs, lncRNAs are transcribed by RNA polymerase II and undergo post-transcriptional processing processes like splicing, 3′-end polyadenylation, and 5′-end capping. In addition to these, three other processing mechanisms contribute to the maturation of lncRNAs (Goff and Rinn 2015).

Unraveling the landscape of ncRNA that regulate different cancer types has become a critical focus in precision oncology research today (Hamdy et al. 2024a, 2024b; Rizk et al. 2024).

### Long intergenic non-coding RNAs

Long intergenic non-coding RNAs (lincRNAs) are a type of lncRNAs, along with intronic lncRNAs, antisense lncRNAs, and promoter-associated long RNAs or enhancer RNAs (eRNAs) (Eldash et al. 2023). Over 50% of lncRNAs are lincRNAs (Kozłowska et al. 2021). LincRNAs are lncRNAs that do not overlap any protein-coding regions and are found in between coding genes. During infection, lincRNAs carry out physiological functions such as inflammation. Numerous cellular processes depend on lincRNAs, which express themselves differently in different tissues (Chen and Shan 2020). Encompassing gene expression regulation, when lincRNA overexpression or mutation disrupts the regulation of gene expression, lincRNAs play a pathogenic role in the formation of cancer (Louca and Gkretsi 2022).

### Implications of lncRNAs with HCC

The pathophysiology, proliferation, apoptosis, differentiation, and tumor growth of HCC are thought to be significantly regulated by lncRNAs (Khan and Zhang 2022a). Tumors can occasionally exhibit aberrant expression of lncRNAs (Wang et al. 2021). They may act as drivers of tumor suppressors or oncogenes. In contrast to genes that code for proteins, tumor-specific lncRNA alterations exist in HCC. Certain lncRNAs in HCC act by chromatin regulation and modification, transcriptional activation, interaction with mRNAs, and sponging microRNAs (miRNAs), all of which have a significant impact on the progression and metastasis of HCC (DiStefano and Gerhard 2022; Hammad et al. 2023). Furthermore, lncRNAs have important roles in determining a tumor’s response to chemotherapy and radiation (Peng et al. 2018). Other mechanisms of lncRNA action are by mediating variables of the oncogenic signaling pathways such, as catenin, Hippo kinase, Wnt, Janus kinase (JAK)/signal transducers and activators of transcription (STAT), and PI3 K/AKT (Chen et al. 2019; Yang et al. 2022).

## The importance of lincRNAs in HCC

HCC represents one of the most common and life-threatening forms of hepatic malignancies worldwide (Llovet et al. 2021). This relies on the fact that it has a complex multifactorial pathogenesis which involves diverse factors including genetic, epigenetic, and environmental ones which interact together yielding this unique form of malignancy (Armandi et al. 2024). This complex pathology may explain, at least partly, the lack of an effective therapeutic modality for the amelioration of this type of cancer (Zheng et al. 2024).

LincRNAs are autonomously transcribed RNA fragments that have a length of more than 200 nucleotides and lack sequence conservation (Mattick et al. 2023). After being transcribed, they undergo post-transcriptional modification and are located in both the nucleus and the cytoplasm (Gao et al. 2020). Being a crucial player in the genomic dynamics, lincRNAs were reported to be incriminated in the pathogenesis of a wide range of malignant neoplasms, including HCC (Khan and Zhang 2022b). This may be due to their ability to promote the aggressive phenotypes of HCC via modulation of DNA methylation and modification of histone deacetylases, with the net result of affecting the patterns of genetic expression in the malignant hepatocytes (Goyal et al. 2024). In addition, the ongoing research efforts have proven that lincRNAs play a fundamental role in the various stages of HCC, including tumor initiation, propagation, distant spread, and the response to therapeutic agents (Liang et al. 2024).

### Role of lincRNAs in HCC initiation

**Fig. 1:**
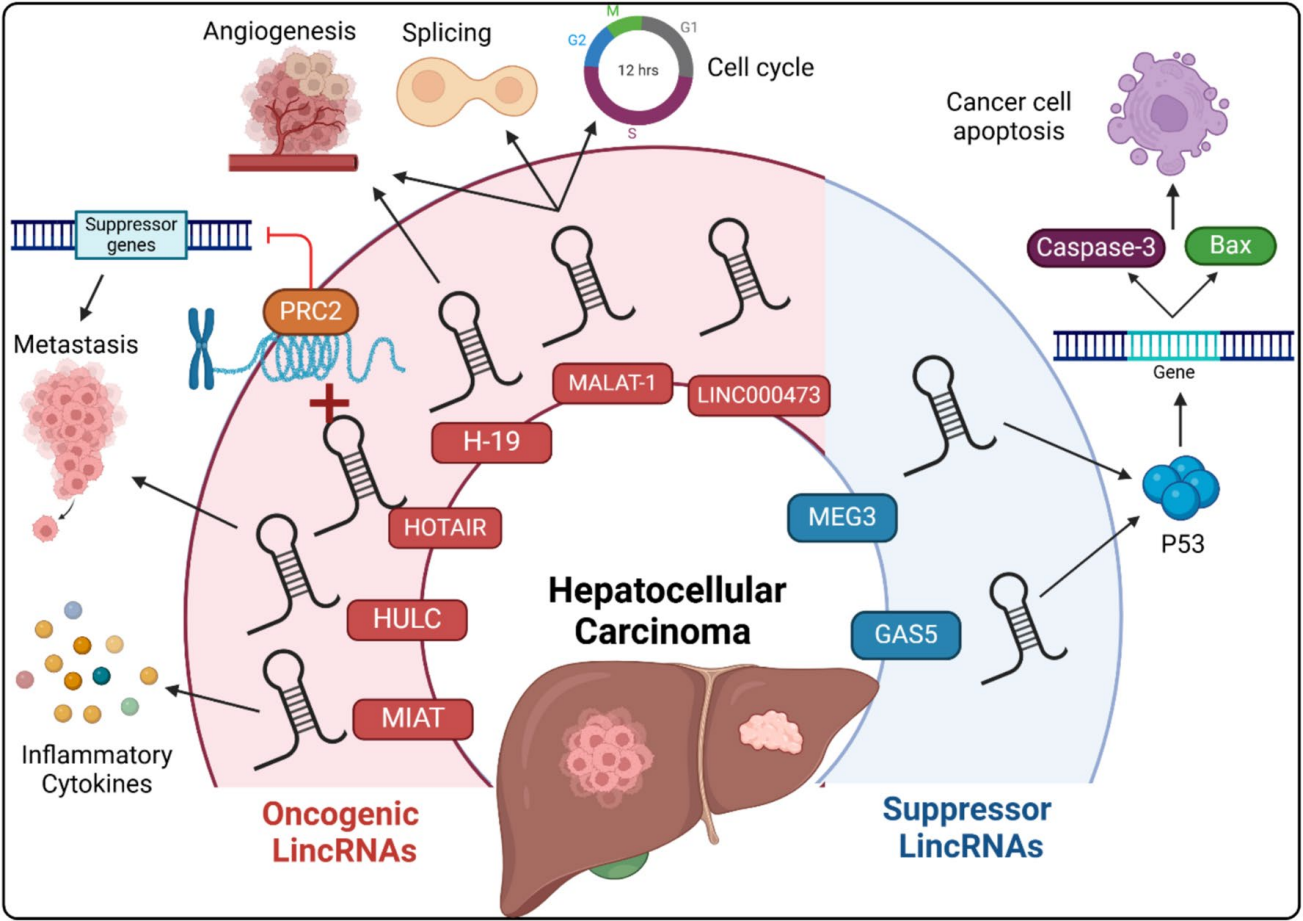
The mechanism of the oncogenic and suppressor lincRNAs involved in HCC. MIAT stimulates the genetic expression of the pro-inflammatory cytokines. HULC facilitates the metastatic spread of the malignant cells. HOTAIR interacts with PRC2, with subsequent inhibition of the tumor suppressor genes and enhancement of metastasis. MALAT1 modulates splicing and the cell cycle. Both MALAT1 and H-19 stimulate angiogenesis. LINC00473 facilitates tumor cell growth and survival. On the other hand, both MEG3 and GAS5 activate p53-mediated transcription and enhance caspase-3 and Bax expression, thereby facilitating cancer cell apoptosis

Recent research efforts were directed towards the exploration of the possible role of lincRNAs in the initiation of HCC (Huang et al. 2020; Liang et al. 2024). They reported that this role may be mediated via interaction of lincRNAs with chromatin-modifying complexes; modulation of the transcription of the genes responsible for cellular proliferation, apoptosis, and metastasis; interaction with RNA-binding proteins; and affection of the stability and translation of mRNA (Wong and Wong 2021). In addition, lincRNAs may bind to miRNAs, thus relieving their inhibitory effect on the oncogenic mRNAs with the net result of initiation of HCC (Khan and Zhang 2022b). LincRNAs can be classified into oncogenic and tumor suppressor types (Lin et al. 2022). The dual roles of lincRNAs in HCC are illustrated in Fig. 1, with clarification of their mechanisms.

#### The link between the oncogenic lincRNAs and HCC initiation

Some types of lincRNAs function as oncogenes, which contribute to the initiation of HCC (Xie et al. 2021). For example, HOX transcript antisense intergenic RNA (HOTAIR) is one of the oncogenic lincRNAs that is found to be overexpressed in cases with HCC (Liu et al. 2021). Its role in the tumor initiation process may be derived from its ability to interact with polycomb repressive complex 2 (PRC2) with subsequent inhibition of the tumor suppressor genes (Zhang et al. 2014). This in turn leads to modification of the chromatin in a way that leads to enhanced expression of the proliferative and invasive phenotypes of HCC, thereby contributing to the initiation of HCC (Huang et al. 2022). Metastasis-associated lung adenocarcinoma transcript 1 (MALAT1) is another example of the oncogenic lincRNAs which is involved in the early phases of tumor development via modulation of splicing and cell cycle, enhancement of cellular proliferation, and inhibition of the pathways related to cell death (Bao et al. 2024). Moreover, MALAT1 enhances the transcription and post-transcriptional modification of various protein complexes, thereby affecting genetic expression and enhancing the signaling pathways related to oncogenesis (Zhao et al. 2019). Another example of the oncogenic lincRNAs is LINC00473 which was proven to have a positive impact on cell growth and survival (Mo et al. 2019). Recent studies have proven that there is a direct relationship between LINC00473 expression and the aggressive behavior of the malignant cells in HCC (Song et al. 2020). Downregulation of LINC00473 was suggested as a promising therapeutic modality for the maintenance of normal cellular homeostasis and prevention of the pathogenic events that lead to initiation of HCC (Zhang et al. 2020).

#### The link between the tumor suppressor lincRNAs and HCC initiation

In the last decade, some types of lincRNAs were identified to have tumor-suppressing properties that hinder the initiation and propagation of HCC, possibly via inhibition of cellular proliferation and enhancement of the apoptotic signals in the hepatic tissues (Khan and Zhang 2022b). For example, maternally expressed gene 3 (MEG3) and growth arrest-specific 5 (GAS5) are tumor suppressor lincRNAs that were reported to be significantly downregulated in cases with HCC (Liu et al. 2019b, Zhang et al. 2022 d). The tumor suppressor properties of MEG3 and GAS5 were suggested to be mediated via activation of p53-mediated transcription and enhancement of the expression of caspase-3 and Bax, which are recognized as key elements of the apoptotic processes (Zhu et al. 2015a; Kaur et al. 2022). Patients with HCC in whom MEG3 or GAS5 are under-expressed were proven to have poor prognosis and increased virulence of the tumor cells (Zhang et al. 2022 d; Lin et al. 2024).

### The role of lincRNAs in HCC progression

Studies concerned with the exploration of the factors that contribute to HCC progression have proven that lincRNAs have a key role in the cellular events involved in the progression and propagation of HCC (Khan and Zhang 2022b). The proposed mechanisms include enhancement of invasion of the surrounding tissues, facilitation of distant spread (Chen et al. 2019), stimulation of angiogenesis, and shaping of both the tumor microenvironment (TME) and the tumor immune microenvironment (TIME) (Elanany et al. 2023).

#### Effect of lincRNAs on tissue invasion and distant metastasis in HCC

Invasion of the surrounding tissues and distant spread is the hallmark of HCC progression and is considered the principal cause of death in patients suffering from HCC (Llovet et al. 2021). Being a main player in the signaling pathways that regulate metastatic spread of HCC malignant cells, lincRNAs may significantly affect the extent of tumor progression and the overall prognosis (Unfried et al. 2021). The most frequently investigated type of lincRNAs in this aspect is the highly upregulated in liver cancer (HULC), which has a direct effect on the epithelial-to-mesenchymal transition, which is the main determinant of invasion and migration of the tumor cells in HCC (Li et al. 2016). Additionally, increased HULC concentration in the hepatic tissues was reported to have an intimate relationship with overexpression of the genes responsible for cellular motility and invasion, which subsequently helps the dissemination of the malignant cells to the distant organs (Zhang et al. 2019a). Another example of lincRNAs that contribute to tumor invasion and metastasis in HCC is HOTAIR, which binds to PRC2, thereby inhibiting the expression of the metastasis-suppressor genes. This consequently initiates a series of events that lead to the disruption of the normal intercellular junctions and facilitate the metastatic spread of the malignant cells (Zhang et al. 2014).

#### Effect of lincRNAs on angiogenesis in HCC

Angiogenesis is a characteristic feature of HCC that is positively correlated with tumor growth, survival, invasion, and distant spread. This may be attributed to the finding that the newly formed blood vessels are the main provider of oxygen and nutrients to the continuously growing malignant cells (Moawad et al. 2020). Growing evidence has revealed that certain types of lincRNAs may act as key regulators of various molecular processes that contribute to angiogenesis in HCC (Tian et al. 2021). For example, H19 is a member of the lincRNA family that facilitates angiogenesis via inhibition of miRNAs (Wang et al. 2023). As a result, miRNA-mediated degradation of the angiogenic factors is suppressed, thereby facilitating the process of new vessel formation and promoting further tumor growth (Tietze and Kessler 2020). Another example of lincRNAs that effectively participates in the process of angiogenesis is MALAT1, which acts via activation and stabilization of hypoxia-inducible factor 1-alpha (HIF-1α), leading to increased expression of the angiogenic factors with enhancement of tumor growth and distant metastasis (Fu et al. 2020).

#### The interaction between lincRNAs and tumor microenvironment

Tumor microenvironment (TME) is a broad term that refers to the different cell types, extracellular matrix proteins, and signaling molecules that interact in harmony in a way that facilitates tumor initiation and propagation (Sas et al. 2022). Myocardial infarction–associated transcript (MIAT) is a type of lincRNA that shows an outstanding ability to shape the TME to make it more suitable for tumor growth, possibly via modulation of the immune processes and affecting the genetic expression of the proinflammatory cytokines (Elmasri et al. 2024). Interestingly, MIAT was reported to regulate the signaling pathways that have a close relationship to the tumor growth and proliferation, such as nuclear factor kappa B (NF-κB)/transforming growth factor beta (TGF-β) signaling (Zhang et al. 2021a).

## Role of lincRNAs in the determination of the response of HCC to the different therapeutic strategies

Due to the complexity of the pathogenic mechanisms that contribute to the development of HCC, the response to therapy is unpredictable and is often confronted with significant resistance (Lohitesh et al. 2018). The ongoing research is directed towards investigation of the possible role that may be played by lincRNAs in determining the response of HCC to the different therapeutic agents (Collins 2015). This may be attributed to the capacity of lincRNAs to modulate the genetic expression and the cellular behavior, thereby determining the extent of the response of HCC to the different therapeutic strategies (Khan and Zhang 2022b).

### Effect of lincRNAs on the resistance of HCC to the chemotherapeutic agents

**Fig. 2:**
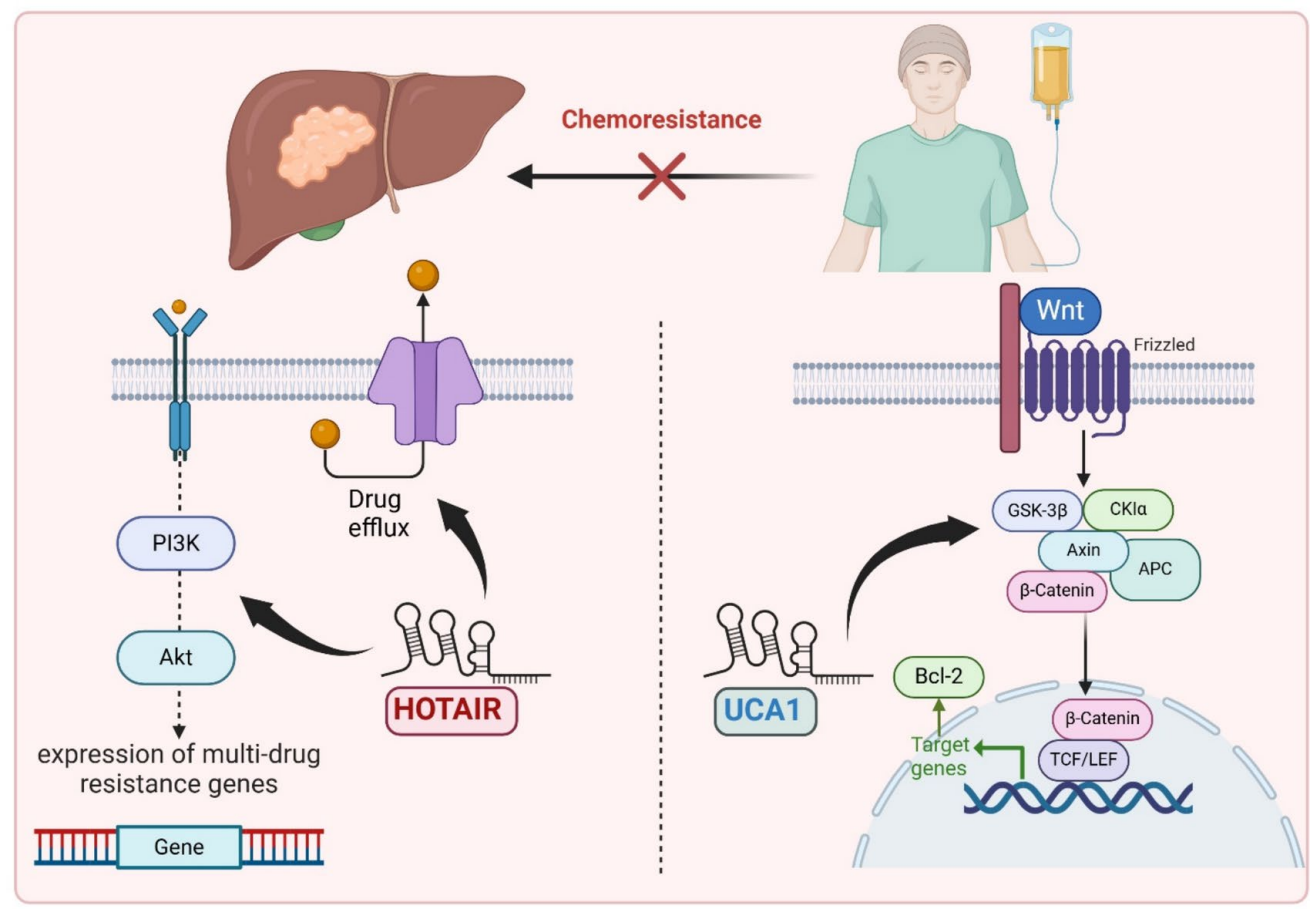
The mechanism of two lincRNAs (HOTAIR and UCA1) involved in chemoresistance in HCC. HOTAIR activates the drug efflux pumps and modulates the PI3 K/AKT pathway in HCC cells, with subsequent enhancement of the expression of multidrug resistance genes, thereby augmenting chemoresistance. UCA1 activates the Wnt/β-catenin pathway, which is critical for tumor cell survival and drug resistance. As a result, β-catenin-mediated Bcl-2 production is enhanced, with subsequent interference with apoptosis of HCC cells

The development of resistance of HCC to the different chemotherapeutic protocols represents an important obstacle that may hinder the efforts of treatment (Lei et al. 2024). Fortunately, a wide range of molecular mechanisms that may contribute to this resistance are affected by members of the lincRNA family (Khan and Zhang 2022b). Of these, HOTAIR stands as the most commonly studied type in the chemoresistance research. The mechanisms by which HOTAIR mediates chemoresistance of HCC include activation of the PI3 K/AKT axis and enhanced expression of the multidrug resistance genes (Rajagopal et al. 2020). This in turn increases the activity of the efflux p-glycoprotein with subsequent extrusion of the chemotherapeutic agents from the malignant cells which significantly reduces their efficacy in HCC treatment (Sun et al. 2021). Urothelial carcinoma–associated (UCA1) represents another example of lincRNAs that has a fundamental role in resistance of HCC to chemotherapy (Qin et al. 2018). This role may be derived from its anti-apoptotic properties which are mediated via activation of the Wnt/β-catenin signaling pathway with subsequent enhancement of the genetic expression of the anti-apoptotic B cell lymphoma antigen 2 (Bcl-2) and repression of the pro-apoptotic mediators (Su et al. 2021). This in turn produces conformational changes in the malignant cells of HCC, making them more resistant to the cytotoxic effects of the chemotherapeutic agents with subsequent treatment failure (Khan and Zhang 2022b). Figure 2 depicts the mechanisms of both HOTAIR and UCA1 in chemoresistance of HCC.

### Effect of lincRNAs on the response of HCC to immunotherapy

In the last decades, immunotherapeutic agents have been introduced as a promising hope for HCC patients, possibly via directing the immune system to target and get rid of the malignant cells (Xing et al. 2021). Due to their proven ability to modulate the TME and regulate the immune response, lincRNAs have emerged as key determinants of the efficacy of immunotherapy in cases with HCC (Gao et al. 2020). The MIAT is a type of lincRNAs that was reported to modulate the immunological infiltration of the TME with subsequent enhancement of the inflammatory cascade and increased secretion of the proinflammatory cytokines (Peng et al. 2020). This in turn has a direct impact on the ability of the immune system to target the malignant cells and the extent to which the immunotherapeutic agents can combat HCC (Elmasri et al. 2024). H19 is another example of immunomodulating lincRNAs that has a direct effect on the genetic expression of the immune checkpoint molecules and cytokines. As a result, significant affection of the number and the activity of the immunocytes within the TME occurs which significantly determines the response of cases of HCC to immunotherapy (Wang et al. 2023).

### Effect of lincRNAs on the response of HCC to targeted therapy

Targeted therapy is a broad term that refers to the different therapeutic strategies that are directed towards specific inhibition of the molecular signaling pathways that mediate tumor initiation and progression, thus avoiding most of the adverse effects of the non-targeted chemotherapeutic agents (Padma 2015). Due to their effect on the genetic expression of a wide range of signaling molecules, lincRNAs may have the ability to modulate the response of HCC to targeted therapy (Laface et al. 2022). For instance, HOTAIR is a lincRNA that was found to regulate the genetic expression of the mediators of drug biotransformation, thereby affecting the individual’s response to targeted therapy (Zhou et al. 2023a). MALAT1 is another example that exerts its effect via modulation of the activity of HIF-1α which controls the process of angiogenesis (Fu et al. 2020). Interference with the HIF-1α pathway was reported to significantly impair the blood supply and the growth of HCC, thereby potentiating the response to the therapeutic strategies for which angiogenesis is the primary target (Dong et al. 2013). Therefore, recent research efforts are directed towards targeting HOTAIR and MALAT1 in an attempt to improve the efficacy and mitigate the resistance of HCC cells to the targeted therapy (Chang et al. 2020; Zhou et al. 2023a). Table 1 summarizes the main types of lincRNAs that have a close association with the pathogenesis of HCC.

**Table 1:** Summary of the roles of the most common lincRNAs that directly impact HCC

LincRNA	Expression	Role in HCC	Ref.
HOTAIR	Upregulated	Oncogene, interacts with PRC2, inhibits TS genes, HCC proliferation, mediates resistance-to-chemotherapy	Liu et al. (2021)
MALAT1	Upregulated	Oncogene, modulates splicing and cell cycle, enhances cellular proliferation, inhibits apoptosis, facilitates angiogenesis, mediates resistance to targeted therapy	Bao et al. (2024)
MEG3	Downregulated	TS, facilitates p53-mediated transcription, modulates apoptosis, inhibits proliferation, invasion	Liu et al. (2019b)
GAS5	Downregulated	TS, activates apoptosis, inhibits proliferation, invasion, and distant metastasis	Zhang et al. (2022d)
LINC00473	Upregulated	Oncogene, microRNA-29a-3p sponge, promote HCC, increases malignant cell aggressiveness	Song et al. (2020)
HULC	Upregulated	Oncogene, stimulates epithelial-to-mesenchymal transition, facilitates invasion, migration	Zhang et al. (2019a)
H19	Upregulated	Oncogene, facilitates angiogenesis and tumor growth, mediates resistance to immunotherapy	Wang et al. (2023)
MIAT	Upregulated	Oncogene, via TME suitable for tumor growth, facilitates tumor proliferation and invasion	Elmasri et al. (2024)

*GAS5* growth arrest–specific 5, *HCC* hepatocellular carcinoma, *HOTAIR* HOX transcript antisense intergenic RNA, *HULC* highly upregulated in liver cancer, *lincRNAs* long intergenic noncoding RNAs, *MALAT1* metastasis-associated lung adenocarcinoma transcript 1, *MEG3* maternally expressed gene 3, *MIAT* myocardial infarction-associated transcript, *miRNAs* microRNAs, *TS* tumor suppressor, *UCA1* urothelial carcinoma-associated, *TME* tumor micro-environment

According to in silico search and analysis, lincRNAs known for their regulation or mechanism of action in HCC are listed in Table 2.

**Table 2:** List of lincRNAs involved in HCC according to in silico search and analysis

LincRNA	Mechanism of involvement in HCC
LincRNA	Regulation by/mechanism of action via
LINC00467	Regulation by GF2BP3 via TRAF5
LINC00460	Sponging miR-485-5p to upregulate PAK1
LINC00261	Suppresses cell proliferation, invasion, and Notch signaling
	Promotes epithelial-mesenchymal-transition and stemness via SMAD3
LINC01133	Activating the PI3 K/AKT pathway to aggravate HCC
LINC00205	Sponge miR-26a-5p facilitating HCC by elevating CDK6
LINC00174	Regulates miR-320/S100 A10 axis
LINC00668	Promotes cell proliferation, migration, invasion, EMT via miR-532-5p/YY1 axis
LINC00461	LINC00461/miR-149-5p/LRIG2 axis regulates HCC
LINC00339	Promotes HCC by miR-1182/SKA1
LINC00472	Suppresses HCC proliferation, migration, invasion through miR-93-5p/PDCD4
LINC01554	Suppresses HCC via downregulating PKM2, inhibiting Akt/mTOR signaling
LINC00665	Promotes HCC via CDK1, BUB1B, BUB1, PLK1, CCNB2, CCNB1, CDC20, ESPL1, MAD2L1, CCNA2
LINC02499	Inhibits HCC cell proliferation, migration, invasion
LINC01093	Downregulated
LINC01139	Promotes HCC progression via upregulating MYBL2, competitively binding to miR-30 family
LINC01134	Linc01134/miR-324-5p/IGF2BP1/YY1 feedback loop mediates HCC progression
LINC00839	Inhibit miR-3666
LINC00520	Upregulates SOX5 to promote HCC by miR-4516

All lincRNAs in the table were retrieved from http://www.rnanut.net/lncrnadisease/index.php/home/search/keyword and https://ngdc.cncb.ac.cn/lncbook/omics/expression. Accessed November 27 th, 2024

## Molecular mechanisms and key signaling pathways of important lincRNAs in HCC

**Fig. 3:**
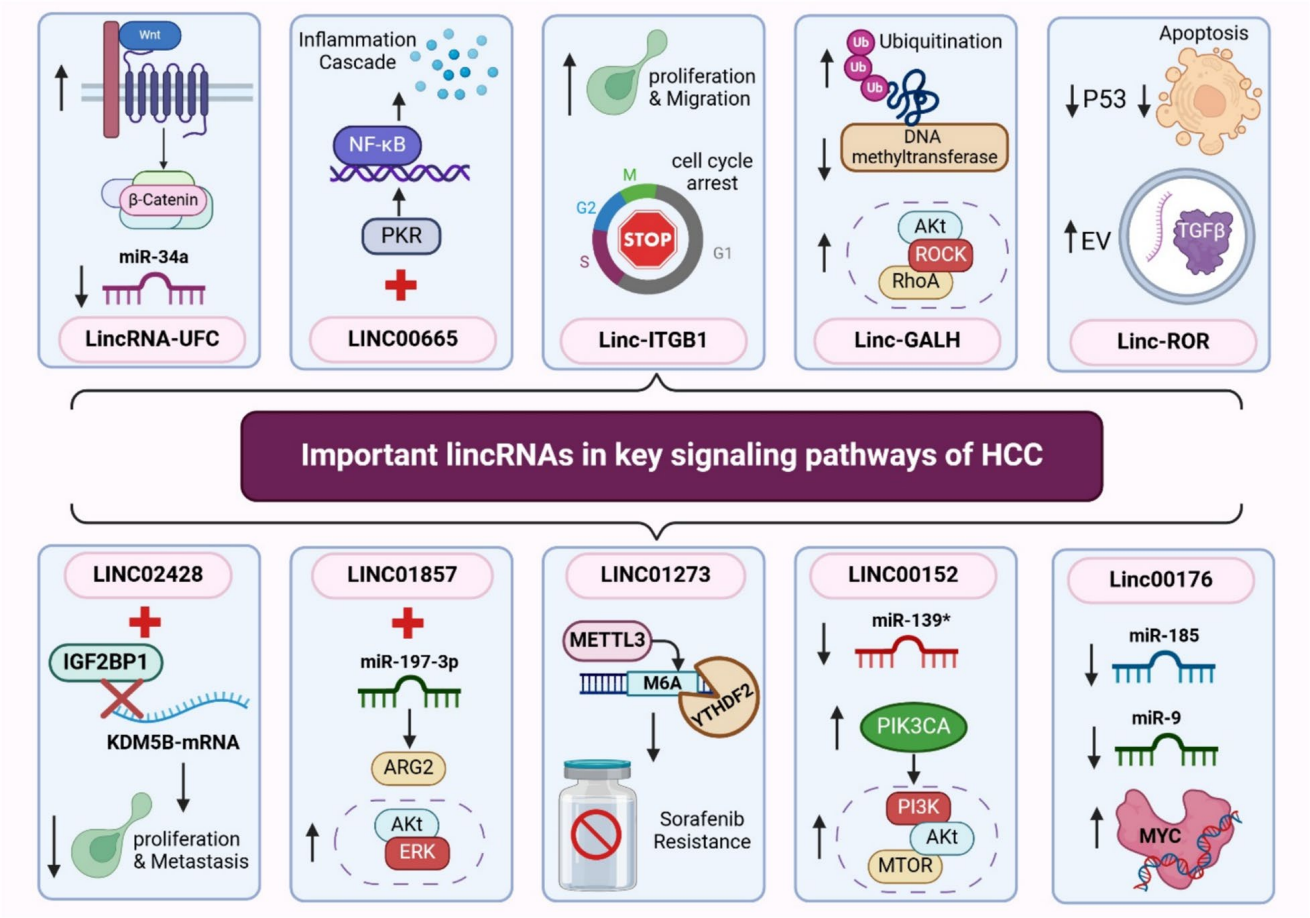
The roles of important lincRNAs in key signaling pathways involved in HCC. The figure illustrates the multifaceted roles of dysregulated lincRNAs, primarily through their interactions with miRNAs, PI3 K/AKT, and NF-κB signaling pathways, in promoting key processes involved in HCC development and progression

While only a few lincRNAs have been fully characterized, lincRNAs are now known to play diverse roles, including influencing chromatin states, regulating transcription, and organizing nuclear structure (Ransohoff et al. 2018). In cancer, lincRNAs exhibit distinct gene expression patterns in primary tumors and metastases. Profiling their differential expression could therefore assist in cancer diagnosis, prognosis, and the identification of potential therapeutic options (Tsai et al. 2011). In HCC, recent findings suggest that lincRNA expression levels are associated with changes in the clinicopathological features of HCC, which can have either promoter or suppressor effects (Liang et al. 2024). The following part highlights the most important individual lincRNAs involved in HCC different signaling pathways, as portrayed in Fig. 3.

### Linc-ROR

The long intergenic non-protein coding RNA, regulator of reprogramming (Linc-ROR), is a stress-responsive lncRNA that is significantly upregulated in HCC, indicating its pivotal role in disease progression (Chen et al. 2018). Linc-ROR facilitates cell-to-cell communication via extracellular vesicle (EV) transfer, enabling stressed cells to impact their microenvironment and activate survival pathways (Wen et al. 2023). Its role in chemoresistance has been observed, with studies showing that sorafenib treatment increases Linc-ROR expression in both tumor cells and EVs (Takahashi et al. 2014). Targeting Linc-ROR with siRNA enhanced early apoptosis in cells treated with sorafenib, evidenced by higher expression of apoptosis- and DNA damage–related genes, including caspase-3/7, caspase 8, and GADD45B, compared to control siRNA. These findings indicate that Linc-ROR plays a significant role in chemoresistance, enriched by TGFβ in EVs. Additionally, Linc-ROR has been shown to inhibit cell apoptosis by repressing p53, with siRNA-mediated knockdown leading to increased p53 activity and demonstrating Linc-ROR’s impact on chemotherapy-induced apoptosis and cell survival (Li et al. 2020d). These insights suggest that targeting Linc-ROR could improve chemosensitivity in HCC by modulating tumor-initiating cells and enhancing the overall chemotherapeutic response.

### Linc-GALH

Another lincRNA with high expression levels in HCC is Gankyrin-associated lincRNA in hepatocellular carcinoma (Linc-GALH), which shows potential as a prognostic marker for HCC. Gankyrin has been shown to activate the AKT pathway by regulating RhoA/ROCK signaling, thereby promoting tumorigenesis and metastasis in HCC (Man et al. 2010). Linc-GALH contributes to the migration and invasion of HCC cells in vitro and enhances their metastatic capability in vivo. Mechanistically, Linc-GALH promotes the degradation of DNA methyltransferase 1 (DNMT1) by facilitating its ubiquitination and increases the expression of Gankyrin in HCC (Xu et al. 2019).

### Linc-ITGB1

Among the lincRNAs with elevated expression in HCC is the lincRNA-integrin subunit β1, known as linc-ITGB1. Research has shown that linc-ITGB1 supports cell proliferation and migration in HCC cells, contributing to HCC progression, likely through a mechanism involving cell-cycle arrest. Consequently, targeting linc-ITGB1 could represent a promising therapeutic approach for HCC (Shang et al. 2017).

### LINC00665

Chronic infection and inflammation are recognized as key drivers of tumor development, particularly in HCC (Refolo et al. 2020). HCC that arises from chronic hepatitis virus infection is a prominent example of cancer linked to both infection and inflammation, commonly occurring in patients with persistent hepatitis B or hepatitis C infections (Park et al. 2007; Bartosch et al. 2009). NF-κB, widely acknowledged as a master regulator of inflammation, plays a significant role in HCC progression (Yu et al. 2018) and has been implicated in the transition from chronic viral hepatitis to liver cancer (Tarocchi et al. 2014; Vescovo et al. 2016).

Interestingly, LINC00665 is an inflammation-induced lincRNA that is overexpressed in HCC and associated with poor patient prognosis (Ding et al. 2020). In HCC cells, LINC00665 is involved in activating NF-κB signaling, which promotes increased cell proliferation in vitro and in vivo. LINC00665 directly interacts with double-stranded RNA-activated protein kinase (PKR), stabilizing PKR by preventing its degradation through the ubiquitin–proteasome pathway and enhancing its activation. PKR activation is known to further stimulate NF-κB signaling, establishing a positive feedback loop (Zamanian-Daryoush et al. 2000). This inflammatory LINC00665/PKR/NF-κB axis may play a critical role in HCC progression, positioning LINC00665 as a promising biomarker and therapeutic target in liver cancer.

### LincRNA-UFC1

The Cancer Genome Atlas indicates that approximately 27% of human HCC cases exhibit a gain-of-function mutation in CTNNB1, which encodes β-catenin. Activation of the Wnt/β-catenin pathway, in synergy with various signaling pathways, promotes HCC development (Xu et al. 2022). Among the lincRNAs involved in HCC progression, lincRNA-UFC1 plays a significant role by directly binding to the mRNA-stabilizing protein HuR, thus increasing β-catenin mRNA and protein levels. LincRNA-UFC1 is more abundant in HCC tissues than in controls and shows a positive correlation with β-catenin levels. Experimental overexpression of lincRNA-UFC1 in HCC cells enhanced cell proliferation and cell-cycle progression while inhibiting apoptosis, contributing to HCC progression. Conversely, miR-34a is negatively correlated with lincRNA-UFC1 levels in HCC tissues, as it destabilizes lincRNA-UFC1 (Cao et al. 2015). This observation aligns with studies that identify miR-34a as a tumor suppressor in liver cancer, where its increased expression is associated with reduced tumor growth in human HCC cells (Wang et al. 2017). Additionally, miR-34a inhibits the Wnt/β-catenin pathway (Zhu et al. 2015b), suggesting that miR-34a may counteract the pro-tumorigenic effects of lincRNA-UFC1 by both targeting lincRNA-UFC1 and downregulating β-catenin. This interaction provides a strong mechanistic basis for understanding the role of lincRNA-UFC1 in promoting HCC.

### Linc00176

MYC is a key oncogenic transcription factor that controls genes related to cell proliferation, metabolism, and immune evasion, contributing significantly to tumor initiation and progression in various cancers, including HCC. In hepatic cancer, MYC and its signaling pathways undergo critical alterations that drive tumor growth, metastasis, metabolic reprogramming, immune modulation, and resistance to treatments, all of which play a major role in HCC progression (Liu et al. 2023).

Linc00176, a MYC target gene, is exclusively overexpressed in HCC and plays a critical role in tumor growth and survival, with its expression correlating with tumor differentiation. MiR-9 and miR-185 are known to have tumor-suppressive roles in HCC (Qadir et al. 2014; Zhang et al. 2015). Linc00176 regulates the expression of genes by acting as a miRNA sponge for the tumor-suppressive miRNAs, miR-9, and miR-185. Although MYC activates miR-9 and miR-185, Linc00176 absorbs these miRNAs, allowing cancer cells to evade their growth-inhibitory effects. Conversely, depletion of Linc00176 releases these tumor-suppressive miRNAs, disrupting the cell cycle and inducing necroptosis in HCC (Tran et al. 2018a).

### LINC00152

The PI3 K/AKT/mammalian target of rapamycin (mTOR) pathway is a key oncogenic signaling cascade that is frequently dysregulated and plays a significant role in HCC development. This pathway is abnormally upregulated in nearly 50% of HCC cases, though the precise mechanisms by which it drives tumor progression remain unclear. Numerous clinical trials have been completed or are currently underway targeting this pathway (Sun et al. 2021). One proposed mechanism of the oncogenic PI3 K/AKT/mTOR signaling pathway is its enhancement of the Warburg effect, which promotes tumor growth and metastasis. Activation of the PI3 K/AKT pathway is also associated with increased cancer aggressiveness and is a significant risk factor for early recurrence and poor prognosis in liver cancer patients. Although research on the PI3 K/AKT/mTOR pathway has led to the development of inhibitors for HCC treatment, the clinical benefits of these inhibitors as single-agent therapies remain limited (Tian et al. 2023).

PIK3 CA encodes the catalytic subunit of PI3 K, which is crucial for regulating processes such as cell proliferation, differentiation, and survival. Mutations in PIK3 CA have been identified in some liver cancer cases. Both preclinical and early clinical findings indicate that activating mutations in PIK3 CA could potentially predict a favorable response to inhibitors targeting the PI3 K/AKT/mTOR pathway (Janku et al. 2014).

The PIK3 CA gene is a key target of miR-139 (Li et al. 2020c). Notably, miR-139 is significantly downregulated in HCC and shows an inverse correlation with disease stage. Patients with elevated miR-139 levels experienced longer overall survival. Overexpression of miR-139 suppresses tumor growth by decreasing cell viability, promoting apoptosis, and reducing colony formation and invasion, whereas its downregulation leads to the opposite effects (Zan et al. 2019). Similarly, miR-139-5p was also reported to be downregulated in HCC, with its overexpression limiting cell viability and invasion, inducing apoptosis, and curbing tumor growth (Li et al. 2019).

Numerous lincRNAs associated with the PI3 K/AKT/mTOR pathway play significant roles in HCC, either through upregulation or through downregulation, demonstrating their therapeutic potential (Wu et al. 2020). Among these, LINC00152 is markedly upregulated in HCC tissues and cell lines and plays a pivotal oncogenic role by influencing the PI3 K/AKT/mTOR pathway. Mechanistically, LINC00152 acts as a miR-139 sponge, relieving its suppression of PIK3 CA, the key target gene. This interaction drives HCC progression by promoting cell proliferation and invasion while inhibiting apoptosis. The oncogenic effects of LINC00152 were confirmed by its knockdown, which diminished these activities; these changes were reversed by anti-miR-139 or PIK3 CA overexpression, further supporting their roles in HCC (Li et al. 2020c). Thus, the LINC00152/miR-139/PIK3 CA axis represents a critical regulatory pathway in HCC, offering deeper insights into hepatocarcinogenesis and positioning LINC00152 as a promising biomarker and therapeutic target for HCC.

### LINC01273

Numerous studies have demonstrated that gene mutations and epigenetic mechanisms, including methylation, chromatin remodeling, and modifications, contribute to the development of HCC (Shi et al. 2023b). Among these, N6-methyladenosine (m6 A) RNA stands out as one of the most prevalent epigenetic modifications of RNA, playing a pivotal role in tumor progression across various cancers, including HCC (Xu et al. 2020; Zhao et al. 2020). The process of m6 A modification is regulated by methylation-related proteins categorized as “writers” (methyltransferases), “erasers” (demethylases), and “readers” (proteins that recognize methylation), which mediate the addition, removal, and recognition of m6 A, respectively (Mei et al. 2023).

Methyltransferase-like 3 (METTL3), a key methyltransferase among the m6 A “writers,” facilitates m6 A RNA modification, which is subsequently recognized by m6 A “readers” like YTH domain family 2 (YTHDF2) (Zhuang et al. 2019). Recent research highlights the significant roles METTL3 plays in various cancer types, both dependent on and independent of its m6 A RNA methyltransferase function (Zeng et al. 2020). For example, HCC progression was reported to be suppressed following METTL3 downregulation (Ma et al. 2024). Additionally, METTL3 was found to be highly expressed in hepatoblastoma tissues and cell lines, correlating with poor prognosis in affected patients. Experimental findings revealed its oncogenic role in hepatoblastoma (Cui et al. 2020).

The lincRNA, LINC01273, was found to be highly expressed in human HCC tissues, particularly in cases resistant to sorafenib, and was associated with poor survival outcomes. Silencing LINC01273 in sorafenib-resistant Huh7-SR and SMMC-7721-SR cells reduced cell viability, colony formation, and DNA synthesis, while restoring decreased m6 A levels in these cells. Mechanistically, LINC01273 acted as a stabilizing reservoir for miR-600, which in turn targeted and reduced METTL3, an m6 A “writer.” Additionally, LINC01273 itself underwent m6 A modification by METTL3, leading to its degradation via YTHDF2, an m6 A “reader.” This feedback loop highlights the critical role LINC01273 plays in developing resistance to sorafenib, suggesting that targeting the LINC01273/miR-600/METTL3 regulatory axis may offer a novel therapeutic approach for HCC patients with sorafenib resistance (Kong et al. 2022).

### LINC01857

Anterior gradient protein 2 (AGR2) has been identified in various types of cancer and shows elevated expression in liver cancer tissues (Obacz et al. 2015). Research by Tsai et al. (2023) suggests that AGR2 influences HCC progression and sorafenib resistance by regulating endoplasmic reticulum stress.

The activation of the extracellular signal-regulated kinase (ERK) and AKT signaling pathways has long been recognized as an indicator of poor prognosis in HCC. Notably, ERK activation in cancerous tissues has also been linked to HCV infection (Schmitz et al. 2008).

LINC01857, another lincRNA overexpressed in HCC, was shown to enhance cell proliferation and suppress apoptosis. LINC01857 competitively binds to miR-197-3p, thereby upregulating AGR2. While miR-197-3p levels are low in HCC, AGR2 is overexpressed. Mechanistic studies indicated that reduced miR-197-3p or increased AGR2 could counteract the suppression of cell growth and induction of apoptosis caused by LINC01857 silencing. Moreover, LINC01857 activated the AKT and ERK pathways through the miR-197-3p/AGR2 axis. These findings suggest that LINC01857 promotes HCC progression by targeting miR-197-3p, upregulating AGR2, and stimulating the AKT and ERK pathways (Bi et al. 2021).

### LINC02428

First of all, insulin-like growth factor (IGF) is related to various cancer types (Furstenberger and Senn 2002; El-Mesallamy et al. 2013b). Specifically, insulin-like growth factor 2 mRNA-binding proteins (IGF2BPs) are reader proteins that interact with mRNA sequences rich in “GGAC” motifs to regulate target gene expression in an m6 A-dependent manner (Huang et al. 2018). The IGF2BP family comprises three isoforms, i.e., IGF2BP1, IGF2BP2, and IGF2BP3, that bind directly to m6 A-modified mRNAs through their K homology domains (Li et al. 2024b). Among them, IGF2BP1 identifies m6 A sites on several METTL3-modified target genes, facilitating the progression of HCC (Fan et al. 2021).

Research by Guo et al. (2021a) revealed that lysine demethylase 5B (KDM5B) regulates YTHDF3 expression by suppressing miR-448, contributing to the increased expression of YTHDF3 in HCC and promoting cancer development.

The lincRNA, LINC02428, was found to have higher expression in normal liver tissues compared to HCC tissues, where it was significantly downregulated. Reduced LINC02428 levels were associated with poor HCC prognosis. Experimental studies demonstrated that overexpressed LINC02428 inhibited HCC cell proliferation and metastasis both in vitro and in vivo. Predominantly located in the cytoplasm, LINC02428 interacts with IGF2BP1, stopping it from binding to KDM5B mRNA, thereby destabilizing KDM5B mRNA. Interestingly, KDM5B binds to the promoter of IGF2BP1 to enhance its transcription. Thus, LINC02428 disrupts the positive feedback loop between KDM5B and IGF2BP1, inhibiting HCC progression. This KDM5B/IGF2BP1 loop plays a key role in the tumorigenesis and advancement of HCC (Du et al. 2023).

## The molecular mechanisms and key signaling pathways underlying the lincRNA-miRNA crosstalk and how this affects HCC progression

**Fig. 4:**
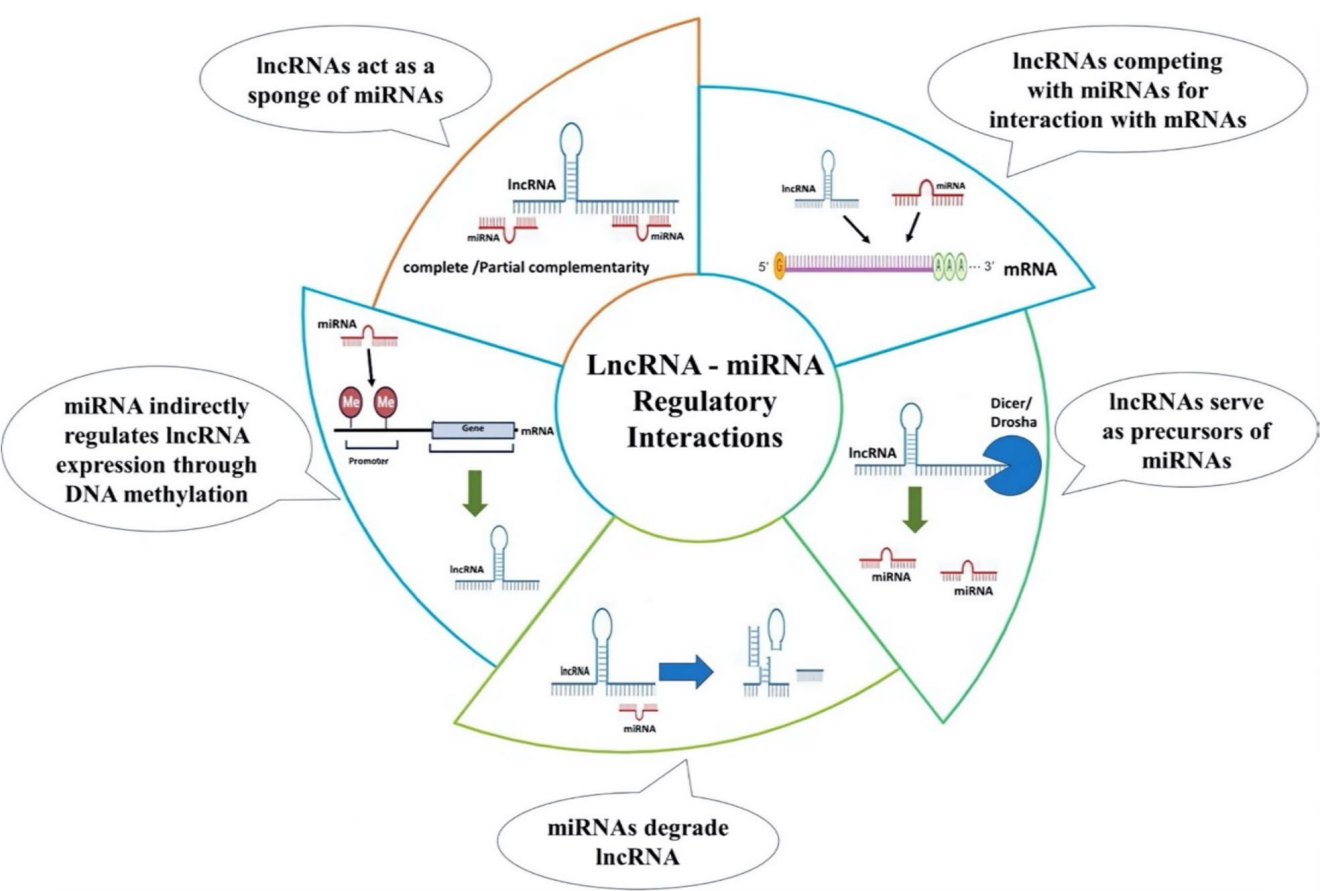
Major regulatory interaction mechanisms between lncRNAs and miRNAs. The main mechanisms of regulation between lncRNAs and miRNAs can be summarized as (1) lncRNAs act as sponges of miRNAs, (2) lncRNAs compete with miRNAs for binding to target mRNAs, (3) lncRNAs can serve as precursors of miRNAs, (4) miRNAs can affect the expression of lncRNA genes through DNA methylation, and (5) miRNAs triggering lncRNA decay

Generally, lncRNAs function by regulating different miRNAs and their target genes, modifying the action of miRNAs and the mRNAs they target. Since miRNAs are regarded as prominent players in HCC development, the complicated interaction between lincRNAs and miRNAs may be crucial in both the development and progression of HCC (Tian et al. 2024). Therefore, it has been proven that lncRNA-miRNA-mRNA interactional networks are a key mechanism behind the carcinogenesis and progression of several cancer types, including HCC. The main mechanisms of regulation between lncRNAs and miRNAs (Yoon et al. 2014; Sun et al. 2020, El-Daly et al. 2023) are summarized in Fig. 4.

In case of HCC, lncRNAs primarily interact with miRNAs through the “sponge effect.” In this process, lncRNAs act as competing endogenous RNAs (ceRNAs), sequestering miRNAs and preventing them from binding to their target mRNAs. This can lead to altered gene expression and subsequent phenotypic changes. LncRNAs can act as ceRNAs by harboring multiple miRNA response elements (MREs) within their sequence. These MREs are complementary to the seed regions of specific miRNAs, allowing the lncRNA to sequester and inactivate the miRNA. This can lead to an increased expression of the target genes of the miRNA, which may have either oncogenic or tumor-suppressive effects (Majoros and Ohler 2007).

In some cases, lncRNAs can bind to miRNAs with perfect complementarity, leading to the formation of double-stranded RNA structures and subsequent degradation of the miRNA-lncRNA complex. More commonly, lncRNAs and miRNAs interact through partial complementarity, primarily involving the seed region of the miRNA. This interaction can lead to translational repression of the target mRNA. The “sponge effect” can be influenced by various factors, including the spatial and temporal expression of lncRNAs and miRNAs (Wang et al. 2013; Sun et al. 2020). Numerous studies have highlighted the lincRNA-miRNA sponge interactions in various cancer types including HCC. For example, in a study, the expression levels of two lincRNAs, HOTAIR and forkhead box protein C1 (FOXC1), in addition to miRNA-1 were assessed in 50 matched pairs of HCC patients and HeG2 cells (Su et al. 2016). In comparison to normal liver tissues and normal cells, the study found that HCC tissues and HepG2 cells exhibited significantly higher levels of HOTAIR and FOXC1 with marked downregulation of miR-1 expression. FOXC1, a well-established transcription factor, plays a critical role in regulating cellular processes. Silencing FOXC1 expression in HCC cells disrupts cytoskeletal organization and significantly inhibits cell proliferation, migration, and invasion (Xu et al. 2012). According to their analysis, FOXC1 directly activates HOTAIR transcription, and HOTAIR sequesters miR-1, resulting in a reduction of its expression. These results imply that the inhibition of miR-1 through interaction with HOTAIR is partially responsible for the carcinogenic role of HOTAIR in HCC.

### Linc-smad7

Linc-smad7 is another oncogenic lincRNA which is upregulated in HCC. The upregulation of Linc-smad7 in HCC patient tissue samples and Huh7 and Hep3B cell lines was associated with poor clinical outcomes and high cellular proliferation, invasion, and epithelial-mesenchymal transition (EMT). By acting as a ceRNA, Linc-smad7 upregulates its target gene, sirtuin 6 (SIRT6), by sequestering miR-125b. As a tumor suppressor, SIRT6 promotes apoptotic cell death, eliminating damaged cells and preventing their uncontrolled proliferation (Vanessa et al. 2017). These results imply that Linc-smad7 is a key player in the development of HCC and could be a therapeutic target (Han et al. 2020).

### LINC01124

LINC01124 was upregulated in HCC tissues and cells (Sun et al. 2022a). It was reported that LINC01124 sponges miR-127-5p which directly targets Forkhead Box Protein O3 (FOXO3) in Hep3B HCC cells. As a member of the forkhead box class O transcription factor family, FOXO3 plays a pivotal role in various cellular processes. In HCC, aberrantly high levels of FOXO3 have been implicated in promoting tumorigenesis and progression. This aberrant activation of FOXO3 contributes to multiple tumor processes (Li and Jiang 2020). Furthermore, inhibition of miR-1247-5p or overexpression of FOXO3 effectively reversed the effects of LINC01124 silencing on the malignant phenotype of HCC cells. These findings demonstrate that LINC01124 exerts a tumor-promoting role in HCC by acting as a ceRNA for miR-1247-5p, thereby upregulating FOXO3 expression (Sun et al. 2022a).

### LINC01194 and LINC00152

LINC01194 and LINC00152 are both oncogenic lincRNAs implicated in the progression of HCC. LINC01194 is overexpressed in HCC tissues and cells, where it reduces apoptosis and promotes migration and proliferation. This action is influenced by acting as a sponge for miR-655-3p, reducing its availability and activity, which in turn affects the expression of its target gene SMAD family member 5 (SMAD5). As a critical mediator in cellular signaling pathways, SMAD5 has been implicated in the development and progression of multiple cancer types (Liu et al. 2022). Similarly, LINC00152 is upregulated through an epigenetic mechanism, acting as a sponge for miR-143a-3p to prevent it from suppressing pro-tumorigenic genes like KLC2. The LINC00152- miR-143a-3p-KLC2 axis enhances HCC cell proliferation, migration, and clonogenicity, and higher KLC2 expression correlates with poorer patient survival (Pellegrino et al. 2022). These findings identify both lincRNAs as potential therapeutic targets in HCC.

Clinical tissue samples of 40 HCC patients and Huh-7 HCC cells both overexpressed LINC01194. The silencing of LINC01194 increased apoptosis in HCC cell lines while inhibiting migration and proliferation. The authors also found that miR-655-3p is significantly downregulated in HCC and that it may bind to LINC01194. The overexpression of miR-655-3p increased the percentage of apoptotic cells while suppressing HCC cell invasion and migration by upregulating its target gene SMAD5. Therefore, by reducing the expression of miR-655-3p, LINC01194 enhanced the development of HCC and could be a suitable therapeutic target for patients with HCC (Liu et al. 2022).

Researchers also discovered that LINC00152 expression is upregulated in HCC due to an epigenetic mechanism. It has been established that LINC00152 sponges miR143a-3p in human HCC cell lines, preventing it from binding to its target genes, such as Kinesin Light Chain 2 (KLC2). KLC2 has been shown to promote tumor growth in human cancer cells. KLC2 was found to be a key mediator supporting the pro-tumorigenic effects of upregulated LINC00152. LINC00152 and KLC2 were also shown to co-express, and a higher expression of KLC2 was linked to a lower patient survival rate. In vitro, KLC2 facilitated cell migration, clonogenicity, and proliferation. So, in human HCC, the LINC00152-miR-143a-3p-KLC2 axis might be a therapeutic target (Pellegrino et al. 2022).

While many studies have investigated the role of lncRNAs as ceRNAs that regulate miRNA activity, the opposite process—where miRNAs directly target lncRNAs—has been observed but studied less extensively. In the context of HCC, the interaction between lincRNA-UFC1 and miR-34a provides a compelling example of such a regulatory mechanism. Researchers have demonstrated that lincRNA-UFC1 is upregulated in HCC and is associated with poor prognosis. Overexpression of lincRNA-UFC1 promotes cell proliferation, inhibits apoptosis, and accelerates tumor growth in xenograft models. Mechanistically, lincRNA-UFC1 interacts with the RNA-binding protein human antigen R (HuR), which is an RNA-binding protein that plays a crucial role in post-transcriptional gene regulation by binding to specific sequences in mRNA molecules. This interaction leads to increased stability of β-catenin mRNA and subsequent activation of the Wnt/β-catenin signaling pathway. Interestingly, lincRNA-UFC1 is also a target of miR-34a. By binding to lincRNA-UFC1, miR-34a can destabilize this lncRNA, thereby reducing its oncogenic potential. This intricate interplay between lncRNA-UFC1 and miR-34a highlights the complex regulatory mechanisms that govern lncRNA-miRNA interaction (Cao et al. 2015).

Table 3 provides a comprehensive overview of emerging research investigating the intricate interplay between lincRNAs and miRNAs in the context of HCC. These studies highlight the multifaceted roles of lincRNAs in regulating several signaling pathways involved in HCC development and progression by acting as competing endogenous RNA to sponge miRNAs.

**Table 3:** Overview of lincRNAs, their targeted miRNAs, and associated pathways in HCC pathogenesis

LINC RNA	Interacting miRNA(s)	Molecular mechanism	Key signaling pathway(s)	Effect on HCC progression	Ref.
LINC-SMAD7	miR-125b	ceRNA sponge miR-125b, leading to upregulation of SIRT6	ERK1/2 signaling pathway	Promotes cell proliferation, migration, invasion, EMT	Han et al. (2020)
SNHG16	miR-195	ceRNA sponge miR-195, leading to upregulation of target genes	SNHG16/miR-195/Ki67, matrix metalloproteinase (MMP) 2 and MMP-9 axis	Enhances cell proliferation, invasion	Xie et al. (2019)
LINC00176	miR-9, miR-185	ceRNA sponge miR-9 and miR-185, leading to upregulation of target genes	Linc00176/miR-9 and miR-185/Ki67, Kinesin Family Member 14 (KIF14), Kinetochore Protein 1 (KNL1), and Spindle Pole Body Component 25 (SPC25) axis	Promotes cell proliferation, survival	Tran et al. (2018a)
LINC01124	miR-1247-5p	ceRNA sponge miR-1247-5p, leading to upregulation of FOXO3	LINC01124/miR-1247-5p/FOXO3 axis	Promotes cell proliferation, migration, invasion	Sun et al. (2022a)
LINCUFC1	miR-34a, lincRNA-UFC1 is also a target of miR-34a., miR-34a can destabilize LINCUFC1 reducing its oncogenic potential	ceRNA sponge miR-34a, leading to upregulation of β-catenin	Wnt/β-catenin	Promotes cell proliferation, inhibits apoptosis	Cao et al. (2015)
LINC00152	miR-143a-3p	ceRNA sponge miR-143a-3p, leading to upregulation of KLC2	LINC00152-miR-143a-3p-KLC2 axis	Promotes cell proliferation, clonogenicity, migration	Pellegrino et al. (2022)
LINC01194	miR-655-3p	ceRNA sponge miR-655-3p, leading to upregulation of SMAD5	Transforming growth factor-beta/SMAD signaling pathway (TGF-β/SMAD)	Promotes cell proliferation, migration, invasion	Liu et al. (2022)
LINC00665	miR-186-5p	ceRNA sponge miR-186-5p, leading to upregulation of MAP4 K3	INC00665/miR-186-5p/MAP4 K3 axis	Promotes cell proliferation, inhibits apoptosis	Shan and Li (2019)
LINC00467	miR-509-3p	ceRNA sponge miR-509-3p, leading to upregulation of platelet-derived growth factor receptor alpha (PDGFRA)	LINC00467/miR-509-3p/PDGFRA axis	Promotes cell proliferation, invasion, inhibits apoptosis	Li et al. (2021b)
LINC01488	miR-124-3p, miR-138-5p	ceRNA sponge miR-124-3p and miR-138-5p, leading to upregulation of vimentin	LINC01488/miR-124-3p and miR-138-5p/vimentin axis	Inhibits cell proliferation, metastasis	Lin et al. (2020)
LINC01006	miR-194-5p	ceRNA sponge miR-194-5p, leading to upregulation of cell adhesion molecule 1 (CADM1)	LINC01006/miR-194-5p/CADM1 axis	Promotes cell proliferation, migration, invasion	Sun et al. (2022b)
LINC01006	miR-433-3p	ceRNA sponge miR-433-3p, leading to upregulation of Chromobox Protein Homolog 3 (CBX3)	LINC01006/miR-433-3p/CBX3 axis	Promotes cell proliferation, migration, invasion	Song et al. (2021)
LINC00667	miR-130a-3p	ceRNA sponge miR-130a-3p, leading to upregulation of androgen receptor (AR)	Androgen signaling	Promotes cell proliferation, migration, invasion	Qin et al. (2021)
LINC01093	miR-96-5p	ceRNA sponge miR-96-5p, leading to upregulation of zinc finger and DNA binding domain containing 5 (ZFAND5)	NF-κB signaling	Promotes HCC pathogenesis	Zheng et al. (2019)
LINC01857	miR-197-3p	ceRNA sponge miR-197-3p, leading to upregulation of AGR2	AKT, ERK signaling	Promotes cell proliferation, inhibits apoptosis	Bi et al. (2021)
LINC00152	miR-139	ceRNA sponge miR-139, leading to upregulation of PIK3 CA	PI3 K/AKT signaling	Promotes cell proliferation, migration, invasion	Li et al. (2020c)
HOTAIR	miR-1	ceRNA sponge miR-1, leading to its downregulation	FOXC1/HOTAIR/miR-1	Promotes cell proliferation, metastasis	Su et al. (2016)
LINC00152	miR-193a/b-3p	ceRNA sponge miR-193a/b-3p to modulate its target gene, Cyclin D1 (CCND1)	LINC00152/miR-193a/b-3p/CCND1 axis	Promotes cell proliferation	Ma et al. (2018)

## Various SNPs in LINC lncRNAs related to key signaling pathways or implicated in HCC

Genome-wide association studies (GWAS) have identified numerous single-nucleotide polymorphisms (SNPs) associated with increased HCC risk, many of which reside in non-coding regions of the genome. These non-coding SNPs can influence gene expression by altering regulatory elements, such as promoters, enhancers, or miRNA binding sites. By modulating gene expression in a cell-type and tissue-specific manner, these SNPs can contribute to the development of HCC (Hettiarachchi and Komar 2022). Interpreting the functional impact of genetic variants, particularly those identified in non-coding regions, is vital for understanding the underlying mechanisms of HCC development and may reveal potential therapeutic targets or preventive interventions.

SNPs within lincRNAs can significantly impact their function and, consequently, contribute to disease susceptibility, including HCC (Qin et al. 2021). While specific SNPs in lincRNAs directly linked to HCC and their precise impact on signaling pathways are still emerging areas of research, several general mechanisms can be considered (Mo et al. 2022). For instance, SNPs can disrupt or create miRNA binding sites within the lincRNA sequence, affecting its ability to act as a ceRNA and leading to dysregulated gene expression (Mo et al. 2022). Additionally, SNPs can modify the secondary structure of the lincRNA, potentially altering its stability, interactions with proteins, or its ability to bind to other RNAs. Furthermore, SNPs in the promoter region of a lincRNA can influence the binding of transcription factors, thereby affecting its expression levels (Singh and Kumar 2023).

Several studies underscore the significance of genetic variations in HOTAIR, particularly SNPs, in influencing the risk of HCC. A study investigating the association between HOTAIR SNPs (rs12427129 and rs3816153) and HCC risk revealed a significant association between these SNPs and increased HCC risk, particularly when combined with environmental factors such as HBV infection. These SNPs may influence the expression or function of HOTAIR, leading to altered gene regulation and tumorigenesis (Zhang et al. 2019b). Another study focused on the rs920778 SNP within HOTAIR and its impact on HCC risk. The TT genotype of this SNP was associated with a significantly higher risk of developing HCC, especially in individuals with specific lifestyle factors like smoking and alcohol consumption. The TT genotype was linked to increased HOTAIR expression, suggesting a potential mechanism by which this SNP contributes to HCC development (Li et al. 2017). A third study investigated the association between three HOTAIR SNPs (rs17105613, rs12427129, and rs3816153) and HCC risk in a Chinese population. The results showed that SNPs rs12427129 and rs3816153 were associated with an increased risk of HCC in dominant genetic models (Cheng et al. 2021). These studies underscore the crucial role of genetic variations within HOTAIR in influencing HCC susceptibility (Hettiarachchi and Komar 2022). A study identified the rs2844512 G > C variant in LINC01149, which was associated with both facilitated HBV spontaneous recovery and increased HCC risk. Mechanistic investigations revealed that this variant creates a binding site for miR-128-3p, leading to the upregulation of MICA expression through a miRNA sponge effect. This upregulation of MICA can have dual effects; it can promote immune cell activation and lysis of infected cells, but it can also lead to immune exhaustion and tumor evasion (Zhong et al. 2020).

Table 4 lists specific SNPs within lincRNAs such as HOTAIR, LINC01149, and GAS5 that have been associated with increased HCC risk, often through mechanisms involving altered gene expression and immune response modulation. To gain a comprehensive understanding of how lincRNA genetic variations contribute to HCC, further studies are needed to investigate their specific functions. Future research should focus on identifying more SNPs within lincRNA that could be associated with HCC risk, elucidating the exact mechanisms through which these SNPs affect tumor development, and exploring their potential as biomarkers or therapeutic targets. By uncovering these molecular mechanisms, we can pave the way for the development of innovative strategies for the early detection, prevention, and targeted therapy of HCC.

**Table 4:** LincRNA genetic variants and their impact on HCC risk

LINCRNA	SNP	Association with HCC	Functional impact	Ref.
LINC01149	rs2844512 G > C	Increased HCC risk, decreased risk of HBV spontaneous recovery	Alters miRNA binding site for miR-128-3p, affecting MICA expression	Zhong et al. (2020)
LINC00673	rs9914618 G > A	Increased HCC risk in elderly (≥ 60 years), association with lymph node metastasis	Affects LINC00673 expression levels	Yuan et al. (2022)
HOTAIR	rs920778 C > T	Increased HCC risk, especially in drinkers and HBV-positive individuals	Alters HOTAIR expression levels	Li et al. (2017)
HOTAIR	rs12427129 C > T and rs3816153 G > T	Increased HCC risk, interacts with HBV infection	Affects HOTAIR expression levels	Zhang et al. (2019b)
HOTAIR	rs12427129 C > T and rs3816153 G > T	Interacts with HBV infection to influence HCC risk	Affects HOTAIR expression levels and interaction with HBV	Cheng et al. (2021)
GAS5	rs145204276 deletion	Increased HCC risk, promotes HCC cell proliferation	Alters GAS5 expression and methylation status	Tao et al. (2015)
HULC	rs1041279	Increased HCC risk, interacts with smoking to further increase risk	Affects HULC expression levels	Wang et al. (2018)

## Clinical implications and therapeutic potential of lincRNAs

It has been widely observed that lncRNAs regulate the biological behavior of cancer cells, particularly HCC, and are implicated in various carcinogenesis and progression (Zhang et al. 2022a). In line with the rapid advancement of cancer research and the ongoing innovation of molecular tools, ncRNAs have emerged as one of the most prominent areas of cancer research (Baptista et al. 2021). LincRNAs are a subclass of lncRNAs that primarily reside and operate in the nucleus. Several investigations revealed that lincRNAs participate in the development and advancement of HCC (Lv et al. 2021). Regarding the diagnosis of HCC, it is mainly dependent on image-based technologies, including computed tomography (CT), magnetic resonance imaging (MRI), or alpha-fetoprotein (AFP) testing (Osho et al. 2020; Parra et al. 2023). Regrettably, the vast majority of individuals with HCC are diagnosed only when curative treatment is no longer feasible, despite the improvements in screening. An opportunity to improve outcomes for HCC patients will arise with early diagnosis via finding early diagnostic biomarkers; thus, lncRNAs with their appealing tissue specificity offer an enticing biomarker target (Xie et al. 2021).

Altered expression levels of lncRNAs significantly correlate with the outcomes of HCC and may as well serve as potential prognostic markers (Califf 2018; Yuan et al. 2021; Chen 2022).

AFP has been widely used in clinical practice for many years. AFP had good diagnostic value as a serum biomarker for HCC. Nevertheless, its sensitivity and specificity may be influenced by the assay techniques, patient attributes, and the degree of severity of the hepatic diseases. Also, AFP has limited sensitivity in early detection (Ma et al. 2020; Hanif et al. 2022). Such constraints highlight the importance of identifying signature lncRNA, specifically lincRNAs, patterns for early diagnosis.

Upregulated Linc-ROR possesses an oncogenic mechanism in HCC, as it directly targets oncogene DEPDC1 and sponges miR130a-3p resulting in the increased progression and angiogenesis of HCC. Consequently, targeting Linc-ROR in therapy may augment sensitivity to chemotherapy and boost treatment outcomes for cancer patients. Additionally, due to its correlation with aggressive cancer traits, Linc-ROR is suggested as a possible biomarker for diagnosis and prognosis (Tian et al. 2021).

Moreover, nuclear LINC02551 coupled with DDX24 and diminished its TRIM27-mediated ubiquitination-related degradation. These effects endorsed EMT and promoted HCC progression (Zhang et al. 2022b). The expression of LincRNA ZNF529-AS1 was significantly associated with age, sex, T stage, M stage, and pathological grade of HCC patients. ZNF529-AS1 was substantially correlated with unfavorable prognosis in HCC and its knockdown suppressed cell invasion and migration, as well as the expression of FBXO31 (Ma et al. 2023). LincRNA PRNCR1 was considered a promoter of cancer progression by controlling miR-411-3p/ZEB1 axis (Zhou et al. 2023b).

The hedgehog signaling pathway (Hh pathway) is closely linked to cancer promotion. Interestingly, cytoplasmic DIO3OS was found to disrupt the Hh pathway by acting as miR-328 sponge. DIO3OS levels were found under-expressed in both tissues and cells of HCC (Wang et al. 2020b). LINC00052 was inversely related to SRY-related HMG-box gene 9 (SOX9), a strong oncogene of HCC and a direct relation with miR-101-3p (Yan et al. 2018). Regarding LINC00511, it was among the over-expressed lncRNAs in HCC, where it could modulate the miR-195/EYA1 axis by acting as a competing endogenous RNA and promoting tumor development and progression (Hu et al. 2020). LINC00511 was correlated with invadopodia and enhanced tumor progression (Peng et al. 2021b).

Hypoxia may enhance the expression of lincRNA-p21 within the microenvironment of solid HCC, consequently exacerbating glycolysis and preventing HCC apoptosis and facilitating tumor development (Li et al. 2021a). Over-expressed LINC01133 is correlated with poorer prognosis of HCC as LINC01133 enhanced tumor proliferation and aggressiveness by sponging miR-199a-5p and increasing SNAI1 also by activating ANXA2/STAT3 survival signaling pathway (Yin et al. 2021). Through the NRF1/DPP4 axis, LINC01132 causes HCC growth and acts as an oncogenic driver. The effectiveness of anti-PDL1 immunotherapy in patients with HCC may be improved by silencing LINC01132. It was also linked with poor survival of HCC patients (Zhang et al. 2022c).

LINC02561 enhances HCC cell migration and invasion by regulating the protein N-Myc downstream regulated 1 (NDRG1). Under hypoxic conditions, HIF-1α can bind to LINC02561 promoters, promoting LINC02561 expression. LINC02561 prevents NDRG1 mRNA degradation. Targeting such axis could provide an innovative strategy for therapy (Li et al. 2024a). Table 5 highlights the potential clinical applications of the different aforementioned lincRNAs.

**Table 5:** Dysregulated lincRNAs in HCC and their possible clinical applications

LincRNA	Targets	Function	Over/under-expression	Ref.
Linc-ROR	DEPDC1	Therapeutic target Diagnostic/prognostic	Over-expressed	Tian et al. (2021)
LINC02551	DDX24	Prognostic biomarker	Over-expressed	Zhang et al. (2022b)
ZNF529-AS1	FBXO31	Prognostic biomarker	Over-expressed	Ma et al. (2023)
PRNCR1	miR-411-3p/ZEB1 axis	Therapeutic target	Over-expressed	Zhou et al. (2023b)
DIO3OS	microRNA-328/Hhip axis	Therapeutic target	Under-expressed	Wang et al. (2020b)
LINC00052	SOX9	Therapeutic target	Under-expressed	Yan et al. (2018)
LINC00511	-	Diagnostic/prognostic	Over-expressed	Hu et al. (2020)
LINC01133	ANXA2	Prognostic biomarker	Over-expressed	Yin et al. (2021)
LINC01132	NRF1/DPP4 axis	Therapeutic target Prognostic biomarker	Over-expressed	Zhang et al. (2022c)
LINC02561	NDGR1	Therapeutic target	Over-expressed	Li et al. (2024a)

## Emerging strategies targeting LincRNAs for HCC therapy

**Fig. 5:**
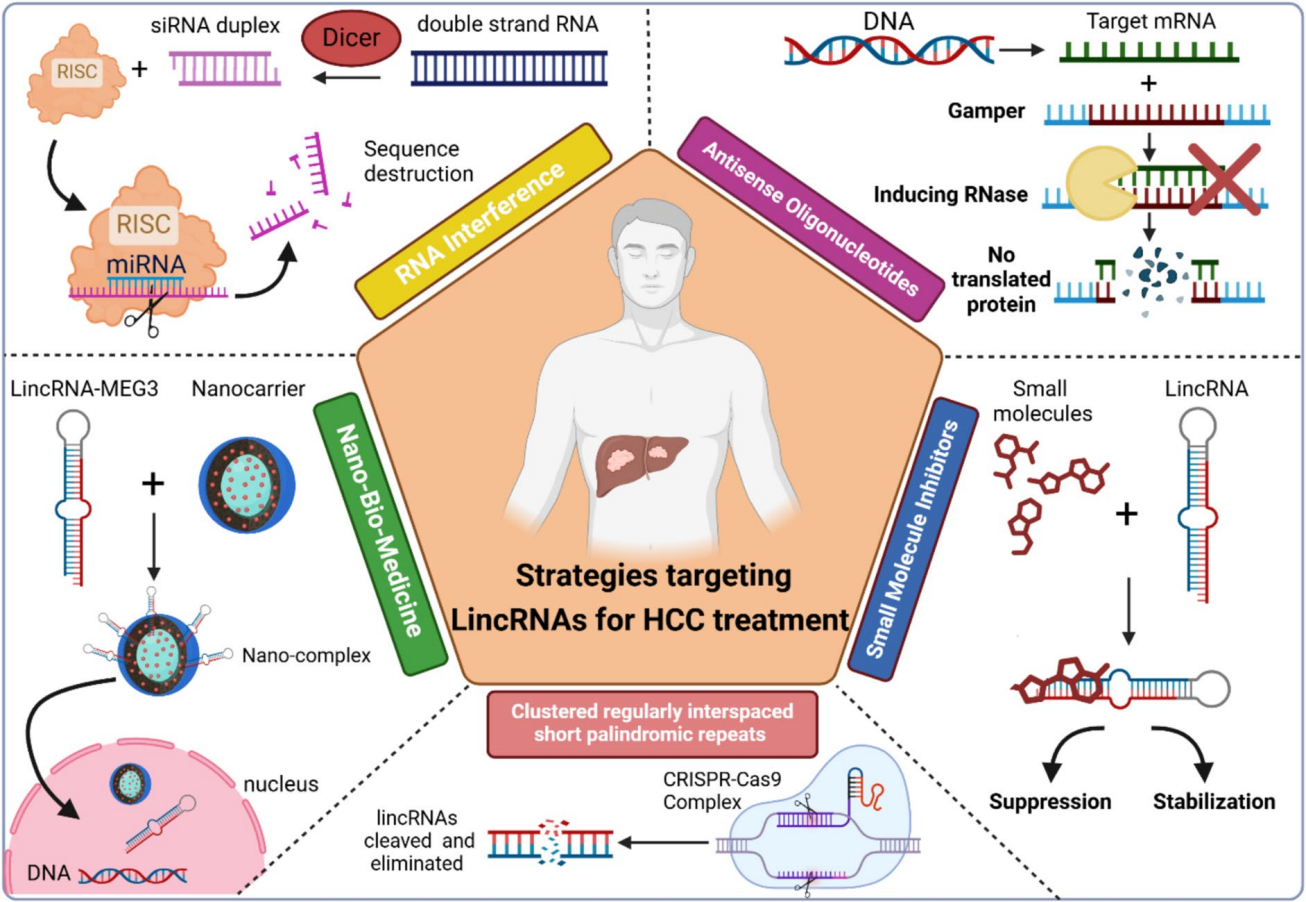
Emerging strategies targeting lincRNAs for HCC treatment. The evolving approaches for HCC treatment targeting lincRNAs including (1) antisense oligonucleotides, (2) RNA interference, (3) small molecule inhibitors, (4) nano-bio-medicine, (5) clustered regularly interspaced short palindromic repeats

Dysregulated cellular mechanisms such as cell proliferation, differentiation, apoptosis, and angiogenesis all have an impact on liver carcinogenesis and HCC progression. LincRNAs have been demonstrated to be epigenetically regulated by or alter the expression of critical HCC-related genes, modulating various biological processes in carcinogenesis and progression in HCC. Because of its complexity and dynamic nature, the HCC microenvironment is an appealing and promising subject for HCC therapeutics to employ combination therapies or localized therapeutic methods (Arun et al. 2018; Li et al. 2020b). Evolving approaches for HCC treatment targeting lincRNAs are depicted in Fig. 5.

### Antisense oligonucleotides

Antisense oligonucleotides (ASOs) are short synthetic nucleic acid strands that bind to complementary RNA, inhibiting the expression of specific genes through mechanisms such as altering RNA splicing or regulating protein translation and RNA stability via RNase H-mediated degradation (Jani et al. 2021). Recent years have witnessed significant advancements in developing biological agents targeting RNA molecules within cells, establishing a foundation for lncRNA-based cancer therapeutics. These therapies fundamentally rely on the inhibition of oncogenic lncRNA or, preferably, on reinstating the expression of tumor suppressor lncRNAs (Mosca et al. 2023). Gapmers, a type of synthetic ASOs, are effective tools for conducting lncRNA loss-of-function research. Gapmers comprise a core DNA segment that triggers RNase H-facilitated RNA degradation, bordered by modified oligonucleotides, including 2′-O-methyl RNA, 2′-O-methoxyethyl RNA, constrained ethyl nucleosides, and locked nucleic acids (LNAs) (Maruyama and Yokota 2020). Nuclear lncRNAs were more efficiently inhibited utilizing ASOs, while cytoplasmic lncRNAs were better inhibited via RNA interference (RNAi) (Lennox and Behlke 2016).

The upregulated Linc-ROR was found to promote chemotherapeutic resistance in HCC, mainly sorafenib, doxorubicin, and adriamycin via various targets (Zhi et al. 2019; Zhang et al. 2021b; Shi et al. 2023a), collectively suggesting the knockdown of Linc-ROR to promote chemotherapy success. LncRNA NR2 F1-AS1 is over-expressed in HCC, particularly the oxaliplatin-resistant tissues, and its downregulation enhanced oxaliplatin susceptibility in HCC (Bu et al. 2014). Moreover, the natural chemical oligo-fucoidan triggered apoptotic signaling in cancer cells, interfering with the expression of lncRNA-Saf and enhancing its anticancer efficacy (Yan et al. 2019).

### RNA interference

RNAi is a highly effective tool for precise, safe, and economical gene silencing. In an innate gene silencing mechanism, long double-stranded RNAs in the cytoplasm are cleaved into small interfering RNAs (siRNAs) that inhibit the expression of homologous genes through complementary RNA degradation. Synthetic siRNAs, which imitate the products of the Dicer enzyme, can be incorporated into this pathway for therapeutic purposes (Hannon 2002; Smekalova et al. 2016). Researchers have developed many treatment techniques based on the RNAi idea to treat HCC by targeting lincRNAs. RNAi employs short double-stranded siRNAs, which facilitate the formation of the RISC complex on the target mRNA, resulting in its sequence-specific destruction (Peng et al. 2021a).

For instance, a study revealed higher levels of muskelin 1 antisense RNA (MKLN1-AS) in HCC tissues, as compared to normal liver tissue, which were correlated with shorter disease-free survival periods and overall survival. The study employed siRNAs and short hairpin RNAs (shRNAs) to knock down MKLN1-AS expression in vitro and in vivo, which resulted in an enhanced anti-cancer effect of lenvatinib (Chen et al. 2022). The silencing of HOTAIR using siRNA can diminish proliferation, migration, and invasion of human HCC cells and impede tumor formation in an HCC mice xenograft model (Yang et al. 2016, Liu et al. 2019a). Despite extensive evaluation of siRNAs for therapeutic purposes across all phases of clinical trials, the primary challenge persists regarding their half-life and delivery mechanisms. For systemic administration, siRNAs must exhibit stability in circulation and resistance to degradation (Mosca et al. 2023).

### Small molecule inhibitors

Modulating the expression levels of lncRNAs with small molecules has become a significant therapeutic approach for managing diseases (Sun et al. 2023). Small-molecule inhibitors, functioning as nucleotide analogs, may interact directly with RNA, including lincRNAs, to modulate disrupted pathways in cancer, thereby demonstrating significant therapeutic potential. They can affect RNA structure and function by stabilizing the conformation of RNA or suppressing RNA–protein interactions, depending upon their methods of action (Alkan et al. 2024). Yet, the field of using small-molecule inhibitors in HCC is premature and requires focus for promising future precision outcomes.

### Nano-bio-medicine (NBM)

Nanotechnology has advanced swiftly, effectively, and reliably, facilitating tailored medicine delivery to lesion areas, potentially minimizing adverse effects and enhancing efficacy (Hamdy et al. 2022a). In patients with HCC, the integration of nano delivery systems with lincRNA treatments presents a significant possibility to enhance therapy options for HCC (Davodabadi et al. 2024; Sokolov et al. 2024). Various nanocarriers, including exosomes, polymer nanoparticles, and microbubbles, have been effectively developed for the delivery of ncRNAs to various diseased cells including cancer cells (Hamdy et al. 2022b).

Nonetheless, each nanocarrier possesses distinct advantages and disadvantages for the effective and safe delivery of nucleic acids (Davodabadi et al. 2024).

The co-delivery approach utilizing PuPGEA/(MEG3 + P53) nanocomplexes exhibits synergistic benefits in inhibiting HCC. The findings indicate that the concurrent delivery of lncRNA and pDNA by polycation nanovectors is a potential approach for cancer treatment (Ren et al. 2016). Moreover, nanoparticles conjugated with MEG3 represent an innovative HCC therapeutic approach as the nanoconjugates exhibited a notable augmentation in tumor histopathology and associated biomarkers (Elzallat et al. 2023).

### Clustered regularly interspaced short palindromic repeats

Clustered regularly interspaced short palindromic repeats (CRISPR) and CRISPR-associated protein 9 (Cas9) knockout have emerged as a prominent method for identifying protein-coding genes essential for cancer cell proliferation and chemotherapeutic drug resistance (Wong et al. 2022), also as a benchmark in genome editing technology offering new strategies for treatment (Noll 2021).

The CRISPR-Cas system has provided a feasible tool for both the deletion or knockdown of protein-coding genes and the inhibition and reduction of non-coding RNA production. Employing the CRISPR-Cas system, lincRNAs may be cleaved and eliminated (Yao et al. 2020). Such approaches could be employed to decrease lncRNA expression such as HULC, HOTTIP, MALAT1, and H19 in vitro and in vivo. Thus, it is seemingly promising to use the CRISPR-Cas system in cancer therapy; however, the off-target effects remain an obstacle when applying the CRISPR/Cas system to therapy (Wang et al. 2020a; Shah and Sarkar 2024).

## Clinical trials

Although clinical trials are of utmost importance to investigate novel treatment avenues in HCC via targeting lncRNAs owing to their clinical relevance as prominent diagnostic/prognostic biomarkers (Luo et al. 2021), yet interventional studies utilizing various lncRNA targeting are still scarce. However, the focus of current trials is to specifically identify and validate HCC-specific biomarkers either for diagnosis or for prognosis (Chan et al. 2024).

## Summary and conclusion

In summary, liver carcinogenesis is closely associated with the dysregulation of various ncRNA types, including lincRNAs. LincRNAs have emerged as paramount regulators that influence multiple cellular and biological processes. As outlined in this review, we have explored the pathogenic implications of lincRNAs in human HCC. Notably, lincRNAs play a dual role in the initiation of HCC. Some lincRNAs (e.g., HOTAIR interacting with PRC2, MALAT1) act as oncogenes, promoting HCC development through different mechanisms. Other lincRNAs function as tumor suppressors, inhibiting HCC initiation and progression. Examples include MEG3 and GAS5, which are downregulated in HCC patients. Their tumor-oncogenic or suppressing activity is linked to the modulation of chromatin-modifying complexes and transcription genes or pathways responsible for cellular proliferation, apoptosis, and metastasis. Understanding these roles offers potential therapeutic targets.

LincRNAs play a crucial role in HCC progression, particularly in metastasis. Mechanisms of metastasis reveal increased expression of HULC and HOTAIR in liver cancer, repressing metastasis-suppressor genes and facilitating the spread of cancer cells. Furthermore, LincRNAs significantly influence angiogenesis in HCC. H19 and MALAT1, for instance, promote angiogenesis by stabilizing HIF-1α and inhibiting miRNAs, leading to increased expression of angiogenic factors. Furthermore, MIAT lincRNA interacts with the TME and alters pro-inflammatory cytokine expression. Therefore, the molecular mechanism of liver carcinogenesis became clearer with a greater comprehension of lincRNA control and dysregulation. The research compiled in this review further provides intriguing examples of the potential applications of lincRNAs as new diagnostic and therapeutic approaches for patients with HCC.

LincRNAs influence the response of HCC to therapy, contributing to unpredictable treatment outcomes and chemoresistance. HOTAIR and UCA1, for example, mediate chemoresistance through PI3 K/AKT and Wnt/β-catenin pathways, leading to drug efflux and reduced efficacy. Understanding these lincRNA-mediated mechanisms is crucial for developing strategies to overcome chemoresistance in HCC. Targeting lincRNAs like HOTAIR and MALAT1 is also a promising approach to improve the efficacy of both immunotherapy and targeted therapy in HCC. MIAT and H19 modulate immune cell infiltration within the TME, impacting the effectiveness of immunotherapy and altering the immune response. LincRNAs like HOTAIR and MALAT1 regulate the expression of drug metabolism enzymes, influencing treatment response.

This review also described the molecular mechanisms and signaling pathways of several lincRNAs in HCC. Linc-ROR, Linc-GALH, Linc-ITGB1, LINC01273, and LINC00665 are upregulated in HCC, promoting cell survival, migration, and chemoresistance, in addition to inducing inflammation and acting as prognostic markers. Other lincRNAs, like LincRNA-UFC1, Linc00176, LINC00152, and LINC01857 exert cross-talks with involved miRNAs in HCC. These lincRNAs utilize diverse mechanisms, often involving interactions with miRNAs signaling pathways (e.g., PI3 K/AKT/mTOR, Wnt/β-catenin, NF-κB), and epigenetic modifications (e.g., m6 A), to influence HCC development, progression, and treatment response.

Furthermore, the current review highlighted the potential role of lincRNAs as promising diagnostic and prognostic targets in HCC, through exhibiting tissue-specific expression patterns, thus offering a potential advantage for early detection. The limitations of AFP’s sensitivity and specificity in early detection underscore the need for lincRNA-based biomarkers. Notably, several lincRNAs have been identified as auspicious therapeutic targets due to their roles in HCC progression and treatment resistance. The pivotal role played by Linc-ROR, LINC0255, ZNF529-AS1, PRNCR1, DIO3OS, LINC00052, LINC00511, lincRNA-p21, LINC01133, LINC01132, and LINC02561 in HCC aligns them as promising candidates for developing novel therapeutic strategies. Other emerging strategies for targeting lincRNAs in HCC therapy include ASOs, RNAi, small molecule inhibitors, NBM, and CRISPR-Cas systems, but further research is necessary to validate these findings and translate them into clinical applications.

In conclusion, lincRNAs hold considerable promise as diagnostic, prognostic, and therapeutic targets in HCC. However, interventional clinical trials directly targeting lincRNAs for therapeutic purposes remain limited. Current clinical efforts are primarily focused on identifying and validating lincRNAs as biomarkers for diagnosis and prognosis. This highlights the need for further research to translate preclinical findings into effective lincRNA-based therapies.

## Future perspectives

Most current research on the role of lincRNAs related to HCC is primarily based on tumor cell lines or tumor xenografts. However, it is essential to recognize that liver cancer possesses unique pathological and physiological characteristics (Rebouissou et al. 2017). Therefore, findings derived from in vitro models should be validated across multiple cell lines and further substantiated through in vivo and clinical studies.

Chronic liver inflammation underlies the majority of HCC cases. Several critical signaling pathways, such as MAPK, PI3 K/AKT/mTOR, Wnt/β-catenin, and JAK/STAT, are intricately involved in HCC pathogenesis (Swamy et al. 2017) and have been associated with circular RNAs. Emerging evidence suggests that lincRNA-circRNA interactions may regulate these pathways (Abaza et al. 2023; Youness et al. 2024), offering promising avenues for clinical diagnosis and therapeutic strategies in HCC (Li et al. 2020a). Nonetheless, the precise mechanisms by which lincRNAs drive the progression from inflammation to neoplasia remain largely unclear.

Beyond its central roles in glucose homeostasis and cholesterol metabolism, the liver is crucial in lipid and lipoprotein regulation (Salama et al. 2023). The increased risk of HCC among individuals with obesity, diabetes, and hepatic steatosis underscores the role of metabolic dysregulation in HCC etiology (Xie et al. 2021). Hence, future research should prioritize identifying lincRNAs that regulate key metabolic processes. This includes lincRNAs potentially associated with myoglobin and thermogenesis (Aboouf et al. 2023), apelin signaling (Aboouf et al. 2015), obesity-related hormones (El-Mesallamy et al. 2013a; Khella et al. 2017), and adipogenesis (Anis et al. 2024). Moreover, exploring the impact of therapeutic agents such as the vildagliptin–pravastatin combination on cholesterol efflux in adipocytes (Mostafa et al. 2016, 2015), particularly in relation to lincRNA expression in HCC, represents a promising research direction.

Although early surgical intervention remains the most effective treatment for HCC, the majority of patients are diagnosed at advanced stages. In recent years, significant progress has been made in tumor immunotherapy, including the development of tumor immunovaccines through reverse vaccinology and immuno-bioinformatics approaches (Bhattacharya et al. 2022). Given HCC’s strong association with chronic inflammation, immunotherapy strategies are particularly relevant. Recent studies suggest that lincRNAs may modulate immune responses in cancer (Guo et al. 2021b), prompting investigations into their potential involvement with immune regulators such as leukocyte-associated immunoglobulin-like receptors (Hammad et al. 2022).

Future studies should therefore focus on the roles of ncRNAs, particularly lncRNAs and lincRNAs, in the context of immunotherapy and immunomodulators (Anwar et al. 2022; Atta et al. 2023; Chen et al. 2023; Chiang et al. 2024). Such insights may pave the way for novel therapeutic strategies tailored to the molecular landscape of HCC.

## Supplementary Information

Below is the link to the electronic supplementary material.

Supplementary file1 (DOCX 20 KB)

## Data Availability

All source data for this work (or generated in this study) are available upon reasonable request.

## Abbreviations

AFP: Alpha-fetoprotein
AGR2: Anterior gradient protein 2
AKT: Protein kinase B
ASOs: Antisense oligonucleotides
Bcl-2: B cell lymphoma antigen 2
CADM1: Cell adhesion molecule 1
Cas9: CRISPR-associated protein 9
CBX3: Chromobox protein homolog 3
CCND1: Cyclin D1
CRISPR: Clustered regularly interspaced short palindromic repeats
CT: Computerized tomography
DNMT1: DNA methyltransferase 1
EMT: Epithelial-mesenchymal transition
ERK: Extracellular signal-regulated kinase
eRNAs: Enhancer RNAs
EV: Extracellular vesicles
FOXC1: Forkhead Box Protein C1
FOXO3: Forkhead Box Protein O3
GAS5: Growth arrest–specific 5
HBV: Hepatitis B virus
HCC: Hepatocellular carcinoma
HCV: Hepatitis C virus
Hh pathway: Hedgehog signaling pathway
HIF-1α: Hypoxia-inducible factor-1-alpha
HOTAIR: HOX transcript antisense intergenic RNA
HULC: Highly upregulated in liver cancer
IGF2BP: Insulin-like growth factor 2 mRNA-binding protein
JAK: Janus kinase
KDM5B: Lysine demethylase 5B
Ki67: Ki-67 protein (a proliferation marker)
KIF14: Kinesin Family Member 14
KLC2: Kinesin Light Chain 2
KNL1: Kinetochore Protein 1
Linc-GALH: Gankyrin-associated lincRNA in hepatocellular carcinoma
linc-ITGB1: Long intergenic non-coding RNA-integrin subunit β1
Linc-ROR: Long intergenic non-protein coding RNA, regulator of reprogramming
lincRNA: Long intergenic non-coding RNA
LNAs: Locked nucleic acids
lncRNAs: Long non-coding RNAs
m6 A: N6-methyladenosine
MAFLD: Metabolic dysfunction–associated fatty liver disease
MALAT1: Metastasis-associated lung adenocarcinoma transcript 1
MAPK: Mitogen-activated protein kinase
MAP4 K3: Mitogen-activated protein kinase kinase kinase 4
MEG3: Maternally expressed gene 3
METTL3: Methyltransferase-like 3
MIAT: Myocardial infarction–associated transcript
miRNAs: MicroRNAs
MKLN1-AS: Muskelin 1 antisense RNA
MMP-9: Matrix metalloproteinase 9
MMP2: Matrix metalloproteinase 2
MRI: Magnetic resonance imaging
mTOR: Mammalian target of rapamycin
NAFLD: Non-alcoholic fatty liver disease
NF-κB: Nuclear factor kappa B
PDGFRA: Platelet-derived growth factor receptor alpha
PI3 K: Phosphoinositide 3-kinase
PIK3 CA: Phosphatidylinositol 4,5-bisphosphate 3-kinase catalytic subunit alpha
PKR: Double-stranded RNA-activated protein kinase
PRC2: Polycomb repressive complex 2
RNAi: RNA interference
shRNAs: Short hairpin RNAs
siRNAs: Small interfering RNAs
SIRT6: Sirtuin 6
SOX9: SRY-related HMG-box gene 9
SPC25: Spindle pole body component 25
STAT: Signal transducers and activators of transcription
TGF-β/SMAD: Transforming growth factor-beta/SMAD signaling pathway
UCA1: Urothelial carcinoma-associated
Wnt: Wingless-related integration site
YTHDF2: YTH domain family 2
ZFAND5: Zinc finger and DNA binding domain containing 5
