# Supplementary material for: LincRNA-miR interactions in hepatocellular carcinoma: comprehensive review and in silico analysis: a step toward ncRNA precision
Source: Naunyn Schmiedebergs Arch Pharmacol. 2025 May 23;398(11):14785–812. doi: 10.1007/s00210-025-04285-7 (PMC12552288; doi:10.1007/s00210-025-04285-7)
Supplement: Supplementary file 1 — Supplementary file1 (DOCX 20 KB) [file 210_2025_4285_MOESM1_ESM.docx]

**Supplementary Table 1S**

| **LINC01348** | Not found | - |
| --- | --- | --- |
| **LINC01608** | Not found | - |
| **LINC02362** | Not found | - |
| **LINC02273** | Not found | - |
| **LINC02475** | Not found | - |
| **LINC01638** | Not found | - |
| **LINC01194** | Not found | - |
| **LINC01089** | Not found | - |
| **LINC01116** | Not found | - |
| **LINC00882** | Not found | - |
| **LINC00958** | Not found | - |
| **LINC01419** | Not found | - |
| **LINC00244** | Not found | - |
| **LINC00161** | Not found | - |
| **GAPLINC** | Not found | - |
| **LINC00662** | Not found | - |
| **LINC00667** | Not found | - |
| **LINC00858** | Not found | - |
| **LINC00857** | Not found | - |
| **LINC00924** | Not found | - |
| **LINC00852** | Not found | - |
| **LINC00844** | Not found | - |
| **LINC00707** | Not found | - |
| **LINC00356** | Not found | - |
| **LINC00355** | Not found | - |
| **LINC00511** | Not found | - |
| **LINC00470** | Not found | - |
| **LINC01503** | Not found | - |
| **LINC01572** | Not found | - |
| **LINC01559** | Not found | - |
| **LINC01612** | Not found | - |
| **LINC02154** | Not found | - |
| **LINC02145** | Not found | - |
| **LINC02870** | Not found | - |
| **LINC02835** | Not found | - |
| **LINC02580** | Not found | - |
| **LINC00992** | Not found | - |
| **LINC00963** | Not found | - |
| **LINC01189** | Not found | - |
| **LINC01152** | Not found | - |
| **LINC01234** | Not found | - |
| **LINC01123** | Not found | - |
| **LINC00673** | Not found | - |
| **LINC00488** | Not found | - |
| **LINC00473** | Not found | - |
| **LINC00638** | Not found | - |
| **LINC01564** | Not found | - |
| **LINC01793** | Not found | - |
| **LINC02882** | Not found | - |
| **LINC02908-201** | Not found | - |
| **LINC00221** | Not found | - |

LincRNAs mentioned “Not found” were retrieved only from <http://www.rnanut.net/lncrnadisease/index.php/home/search/keyword>

and not found or not related to HCC per <https://ngdc.cncb.ac.cn/lncbook/omics/expression> Accessed November 27th, 2024.
